# Engineering of T7 DNA-dependent RNA polymerase with activity at elevated temperature

**DOI:** 10.1371/journal.pone.0353775

**Published:** 2026-07-20

**Authors:** Svenja Hehn, Julia Gutbrod, Maxi Gutjahr, Andreas Marx

**Affiliations:** Department of Chemistry, Konstanz Research School Chemical Biology, University of Konstanz, Universitätsstraße 10, Konstanz, Germany; Kyushu University Faculty of Agriculture Graduate School of Bioresource and Bioenvironmental Sciences: Kyushu Daigaku Nogakubu Daigakuin Seibutsu Shigen Kankyo Kagakufu, JAPAN

## Abstract

Bacteriophage T7 RNA polymerase (T7 RNAP) is a key enzyme for *in vitro* transcription (IVT) and plays a central role in the production of synthetic mRNA for research and therapeutic applications. However, IVT frequently generates double-stranded RNA (dsRNA) as an undesired by-product, which can trigger innate immune responses and compromise mRNA quality. Increasing reaction temperatures reduces dsRNA formation, however, the wild-type T7 RNA polymerase exhibits limited stability under such conditions. Therefore, polymerase variants with enhanced thermotolerance enable more robust transcription at elevated temperatures while minimizing dsRNA generation. To address this limitation, we aimed at obtaining T7 RNA polymerase variants with increased thermotolerance using the Protein Repair One Stop Shop (PROSS) web server. Four crystal structures of T7 RNA polymerase, comprising a promoter complex, an initiation complex, and two elongation complexes were used as input for independent PROSS runs. Mutations shared across all four designs for each PROSS index were then combined to generate multi-structure PROSS Combined Designs (PCDs). In the subset evaluated experimentally, PCD9 retained full-length transcription activity at temperatures up to 48 °C, whereas wild-type T7 RNA polymerase showed strong loss of activity under the same buffer conditions. At 48 °C, PCD9 supported the synthesis of kilobase-length scale transcripts and produced no detectable dsRNA signal in a dot blot assay. In contrast, the wild-type enzyme generated strong dsRNA signals at 37 °C and failed to produce detectable RNA at 48 °C. Additional PROSS variants derived exclusively from the elongation complex structure were inactive at both 37 °C and 48 °C. Together, these results show that multi-structure PROSS design can yield a thermotolerant T7 RNA polymerase with improved performance at elevated temperature and reduced dsRNA byproduct formation. The findings also suggest that restricting stability design to a single structural state may not fully capture the requirements of a highly dynamic enzyme.

## Introduction

T7 RNA polymerase (T7 RNAP), a single subunit DNA dependent RNA polymerase from bacteriophage T7, is a fundamental tool in molecular biology because of its high efficiency and robust activity in transcribing DNA into RNA [1]. Its applications span synthetic biology, controlled gene expression, and the production of RNA for therapeutic purposes, including mRNA based therapeutics [2–4]. However, wild-type (wt) T7 RNAP has reduced performance at elevated reaction temperatures, which has motivated efforts to develop variants that remain activity at elevated temperatures for broader biotechnological use [5,6].

Conducting *in vitro* transcription at higher temperatures can reduce the formation of double-stranded RNA (dsRNA), a major byproduct in *in vitro* transcription reactions [7]. dsRNA can arise through several routes. T7 RNAP shows a limited RNA dependent activity, so short abortive RNA fragments formed during early initiation can anneal and prime complementary RNA synthesis, which generates dsRNA [7,8]. dsRNA can also form when the 3′ end of a full length transcript folds back and self-primes further extension through terminal transferase like activity, which produces loopback dsRNA structures [9]. In addition, promoter independent transcription from DNA ends can generate antisense RNA that hybridizes to the sense strand, which further increases dsRNA formation [10]. Reducing dsRNA can improve product quality and can decrease the need for extensive downstream purification. This is particularly important for RNA therapeutic applications, where dsRNA can trigger undesired innate immune responses [11]. Thermotolerant T7 RNAP variants, i.e., variants that remain catalytically active at higher temperatures than the wild type, are therefore attractive for *in vitro* transcription–based manufacturing at elevated temperatures. They could also facilitate one-pot transcription and cyclization reactions for circular RNA production, for example in protocols based on permuted introns and exons, which require elevated temperatures [12–14]. In addition, thermotolerant T7 RNAP variants are of interest for isothermal, transcription-based amplification methods such as Nucleic Acid Sequence Based Amplification (NASBA) and Transcription Mediated Amplification (TMA) [5,15,16]. These assays are used in rapid viral diagnostics and often operate at temperatures above 40 °C.

Early work on thermotolerant T7 RNAP used random mutagenesis combined with genetic selection, primarily to raise the operating temperature of IVT and isothermal amplification reactions and thereby improve transcription kinetics and amplification of structured and long RNAs. Liao *et al*. applied a two-plasmid screen in *Bacillus stearothermophilus*, originally described by Ikeda *et al*. They identified a serine to proline substitution at position 633 that improved performance at elevated temperature [17,18]. Using a related system, Sugiyama *et al.* described three additional substitutions, S430P, F849I, and F880Y, that similarly enhanced high temperature performance. They further showed that a mutant carrying S430P, S633P, F849I, and F880Y displayed about twelve-fold higher specific activity at 50 °C than wt T7 RNAP [19]. Boulain *et al*. extended this approach by combining random mutagenesis with an *in vivo* suppressor screen in *Escherichia coli (E. coli)*. They identified eleven amino acid substitutions in their screen. Nine mutations enhanced thermostability, five of them had been described previously and four were novel. One mutation primarily improved catalytic activity, and one did not benefit the wt enzyme on its own but increased the activity of certain combination mutants. In that study, thermostability was quantified as T_1/2_, defined as the pre-incubation temperature at which a ten minute heat treatment followed by an activity measurement at 41 °C reduced residual activity to 50% of the activity without pre-incubation. In their best variant, carrying S430P, F849I, F880Y, Q786L, C510R, S767G, and Q744R, additive effects of combined mutations increased T_1/2_ by up to 9.5 °C relative to wt [5].

Structure based and rational strategies have complemented these evolutionary approaches. Sobek *et al.* described the use of X-ray structures to select mutation sites that fill hydrophobic cavities or stabilize surface loops. They reported a triple mutant, V426L, A702V, V795I, showing an approximately 50-fold extension of half-life at 50 °C compared with the wt enzyme [20]. In a subsequent patent, Ong *et al*. reported T7 RNAP variants that produced approximately twice as much RNA as wt at 50–55 °C. They also described fusion constructs that combined RNA polymerases with thermostable DNA binding domains, which prolonged activity at elevated temperatures and slowed inactivation above 56 °C. Notably, their best-performing variant, M20, incorporated 40 amino acid substitutions and retained robust activity above 55 °C [21]. Jain reported rationally designed variants in which mutations predicted to enhance stability while preserving affinity were combined at positions I320, I396, F546, S684, and G788. The best variant, M3, carried I396L, S684A and G788A, and retained about 70% activity at 44.5 °C [22].

Beyond improving stability alone, thermostabilizing mutations can also support additional engineering goals. Meyer *et al*. showed that stabilizing mutations reported by Sugiyama *et al*. can rescue the reduced activity of T7 RNAP variants with altered nucleotide specificity without altering their substrate preference [23,24].

More recently, computational methods have been applied to guide engineering for improved performance at elevated temperatures. He *et al.* used consensus information and folding free energy calculations in combination with the HotSpot Wizard tool to identify sixteen candidate hotspots in the palm domain of T7 RNAP [6]. They selected C530S and G788A and introduced these substitutions into an existing thermotolerant background, M0 (S430P, N433T, S633P, F849I, F880Y) [6]. The resulting M0 + G788A variant supported circular RNA production at 50 °C and reduced dsRNA levels to 20% of those observed for wt T7 RNAP. These results illustrate the value of computational analyses for optimizing performance at elevated temperatures.

Another computational stability design framework is the Protein Repair One Stop Shop (PROSS) web server. PROSS combines multiple sequence alignment with Rosetta based atomistic design to identify mutations outside the active site that are predicted to stabilize a given protein [25]. It first excludes rarely observed amino acid identities based on phylogenetic analysis. It then uses Rosetta modelling to remove destabilizing substitutions and thereby defines a reduced set of single substitutions that are predicted to be stabilizing. In a final step, Rosetta is used to design combinations of these substitutions while accounting for interactions between mutated and wt positions. This approach has yielded highly stabilized enzymes, for example human acetylcholinesterase variants with an increase in thermal stability of about 20 °C and preserved enzymatic properties.

Overall, prior studies show that T7 RNAP thermotolerance can be improved by random mutagenesis, rational design, and more recently by computational approaches. However, many design strategies focus on single structural states and do not explicitly account for the large conformational changes that T7 RNAP undergoes during the transcription cycle. In this study, the PROSS web server was used to design thermotolerant T7 RNAP variants for *in vitro* transcription at elevated temperature by integrating information from multiple crystal structures representing distinct functional states. The aim was to test whether a multi-structure design strategy can generate variants that extend the useful transcription temperature range while preserving catalytic activity and reducing dsRNA byproduct formation.

## Results and discussion

### Multi-structure PROSS design identifies a thermotolerant T7 RNAP variant

**Fig 1:**
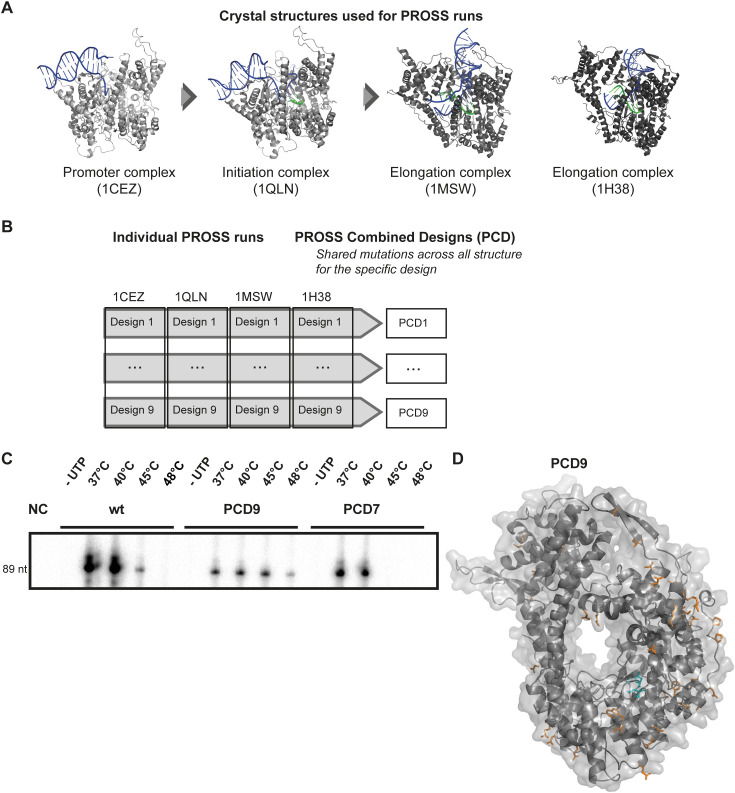
Application of PROSS to T7 RNAP using multiple crystal structures. (A) The four T7 RNAP structural states used for PROSS are shown: promoter complex (PDB 1CEZ), initiation complex (PDB 1QLN), and elongation complex (PDB 1MSW and 1H38). The enzyme is depicted in gray, DNA in blue, and RNA in green. (B) PROSS was run separately on each structure, generating nine designs per structure. For each design number, mutations shared across all four structures were combined to create PROSS Combined Designs (PCDs). (C) Denaturing PAGE of a ^32^P-based *in vitro* transcription assay comparing PCD7 and PCD9 with wild-type T7 RNAP (wt) at 37 °C, 40 °C, 45 °C, and 48 °C. Full-length transcription yields an 89 nt product. NC denotes a no-enzyme negative control, and −UTP indicates a reaction without UTP. (D) Structural context of PCD9 mutation sites (PDB 1H38, DNA template and RNA transcript omitted). T7 RNAP is shown as a light-gray surface with a dark-gray backbone trace. PCD9 mutations are highlighted as orange sticks, and the catalytic residues D537 and D812 marking the active site are shown as cyan sticks.

The PROSS webserver was applied separately to four crystal structures of T7 RNAP that represent promoter (PDB 1CEZ), initiation (PDB 1QLN), and elongation complexes (PDB 1MSW and 1H38) [25–29]. For each structure, nine PROSS designs were obtained. Comparison of the designs across the four structures for each design index revealed that only a subset of substituted positions was shared. When only mutations common to all four structures were retained, the resulting combined designs contained 3 shared substitutions for design 1, 6 for design 2, 7 for design 3, 9 for design 4, 13 for design 5, 18 for design 6, 24 for design 7, 38 for design 8, and 48 for design 9. For experimental evaluation, variants derived from designs 7 (PCD7) and 9 (PCD9) were selected (Fig 1A, B). Designs 1–6 contained only 3–18 shared substitutions and were therefore expected to provide a lower degree of stabilization. PCD7 was selected to represent a combined design with an intermediate mutational load, whereas PCD9 represented the combined design with the highest mutational load. In addition, the number of substitutions in these two designs was consistent with the mutational range of previously reported PROSS variants that showed substantial stabilization [25]. After exclusion of mutations predicted to contact nucleic acids, the final PCD7 and PCD9 variants carried 20 and 43 substitutions, respectively. Full amino acid sequences are provided in the Supporting information (S1 Table. Sequences of variants). Both variants were expressed and purified from *E. coli.*

In a subsequent *in vitro* transcription assay resulting in an 89 nucleotide (nt) transcript, wt T7 RNAP and the two PCD variants were tested at 37, 40, 45, and 48 °C (Fig 1C). At 37 °C, both PCD7 and PCD9 showed lower activity than the wt enzyme. When the temperature was increased, PCD7 lost activity in a pattern similar to wt and did not yield full-length product at 45 °C. In contrast, PCD9 retained the ability to synthesize full-length transcripts at 48 °C under the tested conditions. This identified PCD9 as a thermotolerant variant of T7 RNAP that remained functional at temperatures where the wt enzyme was inactive.

A comparison of the PCD7 and PCD9 sequences revealed 23 positions that remained wt in PCD7 but were substituted in PCD9: A49E, A83K, V114I, V214I, A247T, T256K, K412E, K454E, I482V, E565D, I581L, Q583K, A584R, S606T, V609L, A615T, S661N, S684A, N697K, I718V, K721P, M832F, and S838K. All other substitutions were shared between the two variants. Since only PCD9 exhibited increased thermal tolerance, these 23 substitutions, individually or in combination with shared mutations, likely accounted for the observed difference in activity at elevated temperatures. The mutations in PCD9 are spread throughout the enzyme and are not concentrated at the active site (Fig 1D). Several substitutions in PCD9 have also been reported in previously described thermotolerant variants. For example, S684A is present in the M3 variant reported by Jain [22]. In the patent by Ong *et al.*, residues that match positions mutated in PCD9 include A83K, Y312D, K454P, S495N, A584K, G618Q, M832F, A863P, and A866K [21]. PCD9 contains mutations at all of these sites, but differs at three positions. It carries K454E instead of K454P, A584R instead of A584K, and A866P instead of A866K. When focusing on mutations found in PCD9 but not in PCD7, the overlapping sites are S684, A83, K454, A584, and M832. In addition, some PCD9 substitutions, including I718V and K721P, are located in loop or surface exposed regions. Substitutions in such regions may be more readily tolerated because they are less likely to directly interfere with catalysis or nucleic acid binding. They could influence local flexibility or stabilize peripheral structural elements, but they may also be functionally neutral in the PCD9 background.

Together, these results identify PCD9 as a multi-structure PROSS-derived T7 RNAP variant that retains full-length transcription activity at elevated temperature, whereas PCD7 does not show improved thermotolerance relative to wt T7 RNAP.

### Optimization of buffer conditions to enhance thermotolerance of PCD9 variant

To further improve performance of PCD9 at elevated temperature, buffer conditions were systematically optimized at 48 °C using the 89 nt transcript assay. The concentrations of DNA template, enzyme, NTPs, and Mg^2+^ were varied, and transcription efficiency was assessed by radioactive *in vitro* transcription followed by autoradiographic detection and quantification.

**Fig 2:**
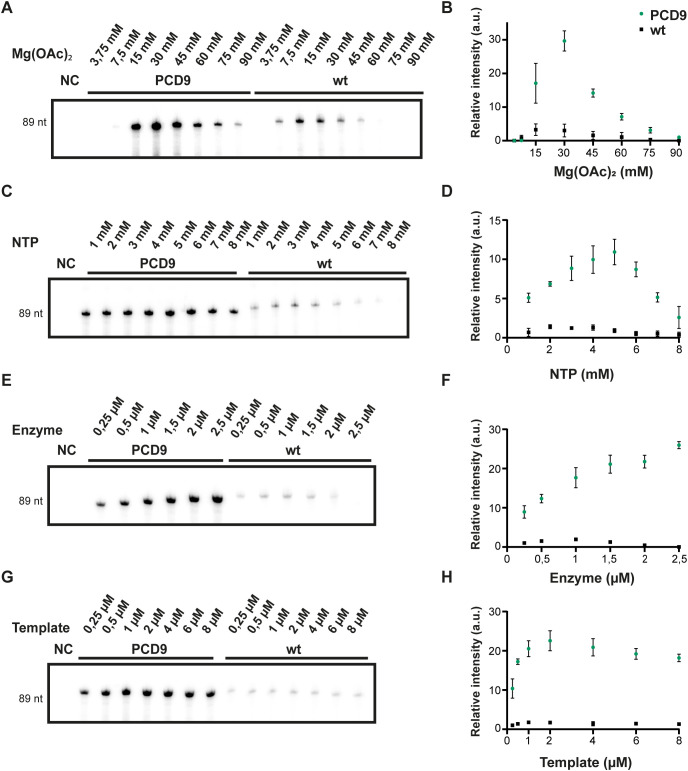
Optimization of transcription conditions for the PCD9 variant at 48 °C. (A, C, E, G) Denaturing PAGE of a ^32^P-based *in vitro* transcription assay testing the effects of Mg(OAc)_2_ (A), NTPs (C), enzyme (E), and template concentration (G) on RNA synthesis. Reactions were run at 48 °C with PCD9 or wild-type T7 RNAP (wt) and included a no-enzyme control (NC). The expected full-length product is 89 nt. (B, D, F, H) Autoradiograph-based quantification shown in arbitrary units (AU). Within each experiment, values were normalized to a chosen reference condition, so results are not directly comparable between experiments. Error bars represent the standard deviation of three independent experiments.

For PCD9, transcription yield increased with rising Mg(OAc)^2^ concentration and reached a maximum at 30 mM, whereas wt T7 RNAP showed only minimal activity under all tested conditions (Fig 2A, B). In these experiments, NTP concentration was kept constant at 3 mM so that only a single parameter was varied at a time, which also influences the observed Mg(OAc)^2^ optimum. When NTP concentration was varied at a fixed Mg(OAc)^2^ concentration of 30 mM, PCD9 exhibited maximal transcription efficiency at approximately 4–5 mM total NTP, with a decrease at higher concentrations (Fig 2C, D). Wt T7 RNAP remained largely inactive across all tested NTP concentrations. Titration of enzyme concentration showed that transcription yield for PCD9 increased up to the highest tested concentration of 2.5 µM, while wt T7 RNAP again displayed low activity at all concentrations (Fig 2E, F). To balance transcription efficiency and enzyme consumption, 1.5 µM PCD9 was selected for subsequent experiments. Finally, variation of template concentration revealed that PCD9 reached maximal activity at approximately 2 µM template, with no further increase at higher concentrations. wt T7 RNAP displayed consistently low activity over the entire range (Fig 2G, H).

Based on these results, and considering resource consumption, 30 mM Mg(OAc)^2^, 4 mM NTPs, 1.5 µM enzyme, and 1 µM template were chosen for further characterization of PCD9 at 48 °C. These optimized conditions were then used to assess the temperature profile of PCD9 in direct comparison with wt T7 RNAP and the M0 + G788A variant.

### Comparison of PCD9 with wt T7 RNAP and M0 + G788A across a temperature series

**Fig 3:**
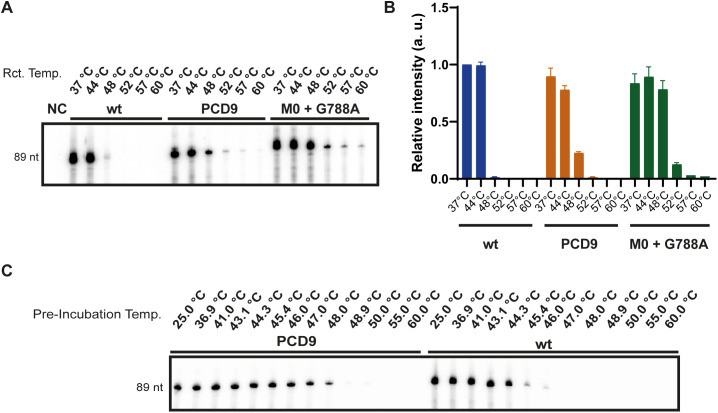
Activity of T7 RNAP wild-type, PCD9 and M0 + G788A across temperatures from 37 to 60 °C. (A) Denaturing PAGE of a ^32^P-based *in vitro* transcription assay with wt T7 RNAP (wt), PCD9, and M0 + G788A at temperatures of 37, 44, 48, 52, 57, and 60 °C. The expected full-length product is 89 nt. NC represents a negative control without enzyme. (B) Quantification of the transcription products shown in (A). Signal intensities were normalized to the wt sample at 37 °C. Blue bars represent wt, orange bars represent PCD9, and green bars represent M0 + G788A. Error bars show the standard deviation from three independent experiments. (C) Thermal inactivation of wt and PCD9. Enzyme variants were pre-incubated for 5 minutes at various temperatures (25.0, 36.9, 41.0, 43.1, 44.3, 45.4, 46.0, 47.0, 48.0, 48.9, 50.0, 55.0, and 60 °C) before the transcription reaction at 37 °C. Denaturing PAGE of a ^32^P-based *in vitro* transcription assay is shown with full-length transcription yielding an 89 nt product.

To place PCD9 in the context of previously described thermotolerant variants, the performance of PCD9, wt T7 RNAP, and the M0 + G788A variant was examined across a temperature range from 37°C to 60°C using the 89 nt transcript assay (Fig 3A, B). The denaturing PAGE analysis showed that wt T7 RNAP showed maximal transcription activity at 37 °C (Fig 3A). Quantification of the full-length product confirmed that RNA yield declined strongly with increasing temperature, and at 48 °C full-length transcription by wt T7 RNAP was nearly abolished (Fig 3B). In contrast, PCD9 maintained substantial activity up to 48 °C and still produced detectable full-length transcript at 52 °C, although at reduced levels (Fig 3A, B). At 57 °C and 60°C, transcription by PCD9 was strongly decreased or undetectable. At 48 °C, full-length product formation for PCD9 was clearly more pronounced than for wt, consistent with previous experiments. The M0 + G788A variant displayed an even broader temperature range. It retained transcription activity at 52 °C and produced weak but detectable product at 57 °C and 60 °C (Fig 3A, B), in line with previous reports that M0 + G788A supports circular RNA production at 50 °C [6]. These data indicate that PCD9 extends the functional temperature range relative to wt, but does not reach the high-temperature performance of M0 + G788A.

To quantify heat-induced inactivation, wt and PCD9 were pre-incubated at elevated temperatures followed by measurement of residual activity at 37 °C. Enzymes were pre-incubated for 5 minutes at temperatures between 25 °C and 60 °C in transcription buffer without NTPs or template. After pre-incubation, a standard reaction at 37 °C was performed (Fig 3C). PCD9 retained transcriptional activity after pre-incubation at higher temperatures than the wt enzyme. A clear reduction in product formation was observed for PCD9 starting at 47.0 °C, and the last detectable full-length transcript was obtained after pre-incubation at 48.0 °C. In contrast, wt activity declined after pre-incubation at 44.3 °C, and only a faint product band was detectable after pre-incubation at 45.4 °C. Because the pre-incubation was performed in the absence of substrates and template, the temperature tolerance inferred from this assay may underestimate the enzyme’s tolerance under active transcription conditions. Binding of template DNA, nascent RNA, NTPs, and divalent metal ions could stabilize the transcription complex and shift the onset of activity loss to higher temperatures. Therefore, this assay should be interpreted as a comparative measure of heat-induced inactivation of the enzyme under non-transcribing conditions rather than as a complete description of stability during active RNA synthesis.

Overall, these results confirm that PCD9 is a T7 RNAP variant with increased thermotolerance compared to the wt enzyme albeit M0 + G788A remains the most thermotolerant variant in this comparison.

### PCD9 supports long transcript synthesis at 48 °C and yields reduced dsRNA levels

To assess performance on a longer template, transcription reactions with wt T7 RNAP and PCD9 were carried out using the pGEM Express Positive Control Template. This template generates two transcripts of 2,346 and 1,065 nt because it contains a T7 terminator whose efficiency has been reported to be approximately 70–80% [30]. Reactions were run at 37 °C and 48 °C, and products were analyzed on a TapeStation.

**Fig 4:**
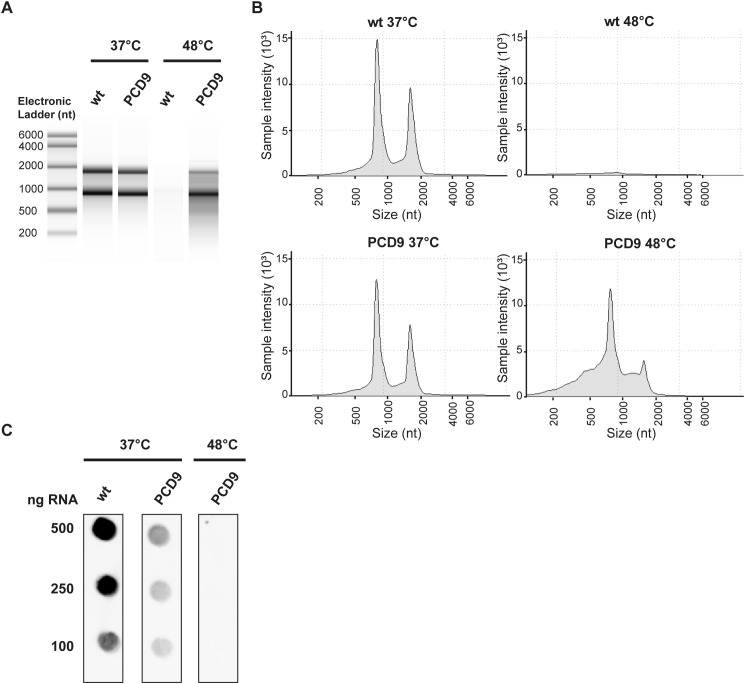
dsRNA production by wild-type T7 RNAP and PCD9. (A) RNA TapeStation gel images of purified *in vitro* transcription reactions performed with wt T7 RNAP (wt) and PCD9 at 37 and 48 °C. Full-length transcription produced transcripts of 1,065 and 2,346 nt using the pGEM® Express Positive Control Template. (B) Electropherograms of the corresponding *in vitro* transcription products shown in (A). (C) dsRNA levels in the purified *in vitro* transcription products from (A) were quantified by dot blot using 500 ng, 250 ng, and 100 ng per spot and detected with SCICONS’s anti-dsRNA monoclonal antibody J2.

At 37 °C, both wt T7 RNAP and PCD9 produced two clear peaks corresponding to the expected transcript lengths (Fig 4A, B). At 48 °C, wt enzyme failed to generate detectable products. In contrast, PCD9 still generated transcripts longer than 1,000 nt. The peak corresponding to the 2,346 nt product was reduced in intensity, and the electropherogram showed a broadened profile with evidence of shorter RNA species (Fig 4B). The gel image also showed heterogenic product formation, which suggested an increased fraction of truncated transcripts (Fig 4A). The presence of truncated transcripts may reflect reduced stability of the elongation complex at 48 °C and an increased tendency for polymerase pausing and premature termination, as described previously for T7 RNAP elongation complexes [31]. In addition, partial RNA degradation during or after transcription may have contributed to the observed shorter RNA species. Sequence specific pausing or termination was not analyzed in the present study, and the fraction of full length RNA could not be quantified precisely from the broadened TapeStation profile. Nevertheless, this broadened RNA profile indicates that elevated temperature transcription with PCD9 did not exclusively generate full-length product. Therefore, while PCD9 enables transcription under conditions where wt T7 RNAP is inactive, the heterogeneous product profile at 48 °C represents a practical limitation for applications that require highly homogeneous full-length RNA. Additional optimization of reaction conditions or downstream purification may be required for such applications. To probe dsRNA content, defined amounts of purified RNA from wt and PCD9 reactions at 37 °C and PCD9 at 48 °C were applied to an Nytran® SuPerCharge nylon membrane and analyzed with a dsRNA-specific antibody (Fig 4C). To enable comparison between samples, equal total amounts of purified RNA were loaded for each condition. Under these normalized loading conditions, wt enzyme at 37 °C gave the strongest dsRNA signal. PCD9 at 37 °C already produced a visibly weaker dsRNA signal. For PCD9 at 48 °C, no dsRNA signal was detectable under the assay conditions. The lack of detectable dsRNA signal for PCD9 at 48 °C, together with its ability to synthesize long transcripts at this temperature, illustrates the potential advantage of a thermotolerant T7 RNAP variant for *in vitro* transcription processes in which minimal dsRNA content is important.

### PROSS variants derived exclusively from the 1H38 elongation complex are inactive

Additional variants were generated from PROSS designs based exclusively on the elongation complex structure (PDB 1H38) to assess the impact of using only a single structural state as design input [29]. After exclusion of residues contacting nucleic acids, designs 1, 3, 5, and 9 contained 58, 71, 95, and 136 substitutions, respectively. All four variants were expressed and purified, which indicated that they were at least sufficiently folded and stable to be produced in *E. coli*.

**Fig 5:**
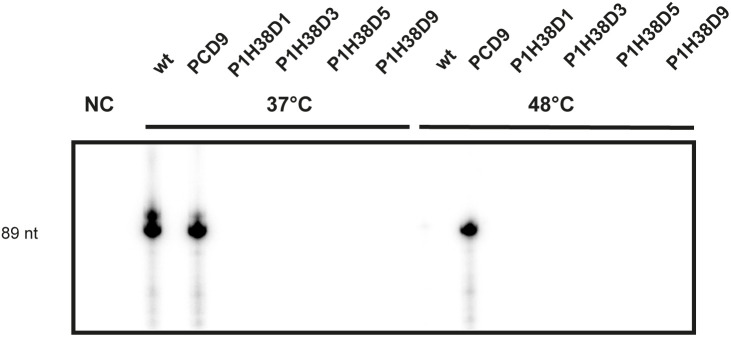
Activity of 1H38 PROSS designs 1, 3, 5, 9 at temperatures of 37 and 48 °C. Denaturating PAGE of ^32^P-based in vitro transcription assay of the 1H38 PROSS designs 1, 3, 5, 9 at 37 and 48 °C in comparison to PCD9 and wt T7 RNAP (wt). Full length transcription yields in a 89 nt transcript. NC is a negative control without enzyme.

Despite successful expression and purification, none of the 1H38-based variants showed detectable transcription activity in ^32^P-based *in vitro* transcription assays at 37 °C or 48 °C. In the same assays, wt T7 RNAP produced full-length product at 37 °C, and PCD9 remained active at 48 °C (Fig 5). The inactivity of these elongation-based designs, suggests that stability design constrained to a single structural state and allowing very high numbers of substitutions can shift the stability-flexibility balance away from the conformational dynamics required for efficient catalysis by T7 RNAP.

## Discussion

This work shows that computational stability design using the PROSS webserver with multiple structural states can generate a thermotolerant T7 RNAP variant that remains transcriptionally active at elevated temperature and produces less dsRNA byproduct. PCD9 carries 43 substitutions that together support full-length transcription at 48 °C under optimized buffer conditions. In contrast to the previously reported M0 + G788A variant, which shows superior high-temperature performance, PCD9 provides a moderate improvement in thermotolerance relative to wt T7 RNAP [6].

PCD9 remains functional on a kilobase-legth scale template at 48 °C and generates substantially less dsRNA than the wt enzyme under the tested conditions. Wt T7 RNAP fails to produce detectable long transcripts at 48 °C and gives strong dsRNA signals at 37 °C, whereas PCD9 already reduces dsRNA formation at 37 °C and shows no detectable dsRNA signal at 48 °C in the dot blot assay. The dot blot analysis was performed with equal total amounts of purified RNA, allowing comparison of detectable dsRNA levels relative to total recovered RNA. However, this normalization does not account for differences in product composition, particularly the broader RNA profile observed for PCD9 at 48 °C. Additional normalization to full-length ssRNA would therefore be required to compare dsRNA formation per full-length transcript more precisely. Notably, PCD9 reduced dsRNA formation already at 37 °C, suggesting that lower dsRNA levels may not be explained by thermotolerance. It could also reflect altered initiation kinetics, elongation behavior, or abortive transcription. However, kinetic parameters such as Km and Vmax were not determined in this study. Therefore, the mechanistic basis for the reduced dsRNA signal remains unresolved and will require dedicated kinetic measurements. Together, these results indicate that PCD9 offers improvements for *in vitro* transcription workflows in which dsRNA byproducts must be minimized, including the production of therapeutic mRNA and circular RNA. M0 + G788A was included as a benchmark in the short transcript temperature series, but not in the kilobase-length transcription and dsRNA byproduct experiments. These experiments were designed to characterize the newly identified PCD9 variant under conditions where wt T7 RNAP is inactive. Therefore, while the data show that PCD9 supports long RNA synthesis at 48 °C and shows reduced detectable dsRNA signal relative to wt under the tested conditions, they do not provide a direct comparison with M0 + G788A in this assay format.

The comparison with PROSS designs based solely on the elongation complex structure highlights a potential limitation of stability design strategies that treat an enzyme as a single static structure. The 1H38-based variants carried up to 136 substitutions and were expressed in soluble form, but they were catalytically inactive at both 37 °C and 48 °C. T7 RNAP undergoes large conformational changes during the transcription cycle, especially during the transition from abortive initiation to processive elongation [26–29]. Extensive stabilization of a single conformational state may restrict the motions required to complete a full catalytic cycle. In contrast, the multi-structure design strategy used for PCD9 required mutations to be compatible with several structural models of T7 RNAP, which likely helped preserve the conformational flexibility required for the full catalytic cycle. This is consistent with the general rationale of multi state design for conformationally dynamic enzymes [32]. At the same time, the consensus based mutation selection used here represents a conservative design strategy. By retaining only substitutions that were compatible with all structural inputs, the workflow may have reduced the risk of stabilizing one conformation at the expense of another conformation required during transcription. However, this criterion may also have excluded substitutions that are neutral in most conformations but particularly beneficial for stabilizing or optimizing a specific state. Such conformation selective mutations were not prioritized in the present workflow and may represent an important additional design space for future T7 RNAP engineering. In addition, the present work only tested PROSS designs derived from a single elongation complex structure (PDB 1H38), and designs based on alternative elongation-state structures or other individual transcription states may yield variants that are both active and thermotolerant. A systematic comparison of PROSS designs generated from different single structures and from multi structure combinations will therefore be important for drawing more robust conclusions about the impact of the design input.

The observation that PCD9 did not reach the high temperature activity of M0 + G788A indicates that PROSS based stabilization of wt T7 RNAP is not sufficient by itself to match variants assembled from previously validated thermostabilizing mutations. M0 + G788A already represents an activity compatible high temperature background, whereas the present PROSS workflow primarily selected substitutions predicted to stabilize the T7 RNAP structure while remaining compatible with several structural models. Thus, the outcome is better viewed as a limitation of the design objective used here rather than as an inherent limitation of PROSS. Future strategies should therefore use established thermotolerant backgrounds such as M0 + G788A as starting points and introduce PROSS predicted substitutions in smaller, spatially or functionally grouped sets. Partial revertant analysis of PCD9 and recombination of selected PCD9 mutations with M0 + G788A would help distinguish substitutions that contribute to thermotolerance from substitutions that are neutral or detrimental for transcription.

Because the present study focused primarily on transcription activity, future studies could use native PAGE and complementary biophysical methods to assess potential folding differences between wt T7 RNAP and the engineered variants and to clarify how the introduced mutations affect protein folding and thermal stability.

## Conclusion

Multi-structure PROSS design yielded PCD9, a T7 RNAP variant with increased thermotolerance that extends the useful transcription temperature window to 48 °C under optimized buffer conditions and reduces dsRNA byproducts when transcribing a kilobase-length scale template. PCD9 is more thermotolerant than wt T7 RNAP but less thermotolerant than M0 + G788A under the same assay and reaction conditions, providing a useful option for *in vitro* transcription workflows operating at moderately elevated temperatures. In contrast, PROSS variants derived from a single elongation complex structure were inactive, indicating that stability design for T7 RNAP may benefit from considering more than one conformational state. PCD9 therefore illustrates both the opportunities and constraints of current computational design strategies and provides a starting scaffold for iterative engineering of T7 RNAP variants that combine enhanced thermotolerance and low dsRNA formation.

## Materials and methods

### General information

T7 RNAP variants were ordered as gene fragments from Azenta. BL21 competent cells were used for expression and XL10 gold cells were used for cloning procedures. Templates for *in vitro* transcription experiments and primers were ordered from Biomers. NTPs were purchased from Carl Roth. RNase inhibitors were obtained from Jena Bioscience.

### PROSS designs

Stabilized T7 RNAP variants were generated using the PROSS web server, based on crystal structures of different enzyme complexes (PDB IDs: 1CEZ, 1QLN, 1MSW, and 1H38) [25–29]. PROSS calculations were performed with default settings except for the following user-defined parameters. The position-specific scoring matrix (PSSM) threshold was set to values greater than zero, and only mutations with calculated energy changes ΔΔG ≤ –0.45 Rosetta energy units were allowed. Homologous sequences used for the multiple sequence alignment were filtered for a minimum sequence identity of 35% and a minimum sequence coverage of 75%. The maximum number of retrieved sequences was limited to 4,000, and the E-value cutoff for BLASTp searches was set to 0.0001. To preserve catalytic function, mutations at residues 537 and 812, which coordinate divalent magnesium ions, were excluded from design. In addition, PROSS predicted substitutions were inspected for direct contacts with the DNA template or RNA transcript using PyMOL and PDIviz, and positions with direct nucleic acid contacts were reverted to the wild type residue. We did not apply a broader distance based exclusion shell around the active site, because this would have removed many positions in structurally dynamic regions that may contribute to stability while still being compatible with catalysis.

For the combinatorial design approach, the outputs of the individual PROSS runs for the four structural models were compared, and only mutations that appeared in all four designs for a given position were retained. If a site was mutated in all four structures but to different amino acids, the residue present in the majority of structures was chosen. In cases where two alternative substitutions occurred with a 2–2 distribution across the four models, one of the two was selected. The resulting consensus mutation sets for each design index were: design 1, 3 mutations; design 2, 6 mutations; design 3, 7 mutations; design 4, 9 mutations; design 5, 13 mutations; design 6, 18 mutations; design 7, 24 mutations; design 8, 38 mutations; and design 9, 48 mutations. From these combined designs, designs 7 and 9 were selected for experimental testing. To avoid perturbing nucleic acid binding, all PROSS-predicted mutations were inspected in PyMOL using the PDIviz plugin, and positions with direct contacts to the DNA template or RNA transcript were reverted to the wt residue. This filtering step reduced the total number of substitutions to 20 and 43 for designs 7 and 9, respectively. Protein sequences with the corresponding mutation sites annotated are provided in the Supporting information (S1 Table. Sequences of variants.). The final coding sequences were generated using the IDT *E. coli* codon optimization tool and synthesized by Azenta, with SphI and HindIII restriction sites incorporated for cloning.

In addition, variants based exclusively on the elongation complex structure (PDB ID: 1H38) were designed using the same workflow. After removal of mutations at nucleic acid-interacting residues, four designs were selected for further analysis, containing 58, 71, 95, and 136 substitutions for designs 1, 3, 5, and 9, respectively. Annotated protein sequences indicating the corresponding mutation sites are provided in the Supporting information (S1 Table. Sequences of variants.). Gene fragments for these 1H38-based variants were synthesized by Azenta and carried the same restriction sites as the combinatorial PROSS designs to facilitate cloning.

### Cloning

Gene fragments and plasmid DNA were digested with SphI-HF and HindIII-HF restriction endonucleases according to the manufacturer’s instructions. Briefly, 0,5 μg of DNA was incubated with 0.5 μL (10 units) of each restriction enzyme for 45 minutes at 37 °C in the appropriate 1x reaction buffer. The restriction enzymes were heat-inactivated at 80 °C for 20 minutes. The resulting DNA was purified by preparative agarose gel electrophoresis or using a reaction Cleanup Kit (Zymo Research). Ligation of the pre-cut DNA insert into the pre-cut DNA plasmid vector was performed by incubating 30 fmol of the vector with a 5-fold molar excess of the insert in the presence of 1 μL (400 units) T4 DNA ligase in 1x T4 reaction buffer in a total reaction volume of 40 μL. Ligation was performed by cycling between 10 °C and 30 °C at 30-second intervals for 12–16 hours. The ligase was inactivated by incubation at 65 °C for 10 minutes and the ligation reactions were stored at −20 °C or used directly for transformation.

### Transformation of chemically competent *E. coli* cells

5 µL of ligation mixture was incubated with 100 μL chemically competent *E. coli* cells (XL-10 gold) for 10 minutes on ice. The cells were heat shocked at 42 °C for 30 seconds and incubated again on ice for 10 minutes. The cell suspension was added to 1 mL of SOC medium and incubated at 37 °C for one hour. 10–100 μL of cells were plated on an agar plate containing the appropriate antibiotics and grown overnight at 37 °C.

### Expression of T7 RNAP variants

*E. coli* BL21 cells were transformed with pGDR11 containing the respective T7 RNAP variant gene. Expression of T7 RNAP variants was performed by inoculating 250 mL to 500 mL LB-medium supplemented with 100 µg/ml carbenicillin with an *E. coli* overnight liquid culture (1:100). Cells were grown at 37 °C and 180 rpm to an OD_600_ of 0.6–0.7 and expression was induced by adding IPTG to a final concentration of 1 mM. After 5 hours of expression, cells were harvested by centrifugation (4000 rpm for 20 minutes, 4 °C) and pellets were stored at −20°C until further use.

### Purification of T7 RNAP variants

Purification of polymerases bearing an N-terminal polyhistidine (6 × His) tag was performed by nickel affinity chromatography using cOmplete™ His-Tag Purification resin (Roche). For this purpose, cell pellets were resuspended in 10 mL T7 RNAP lysis buffer (50 mM Tris-HCl (pH 8), 300 mM NaCl, 0.1% Triton X-100, 0.1 mg/mL Lysozyme) and incubated on ice for 30 minutes. The cells were disrupted by sonication and the lysates cleared by centrifugation (25,000 × g for 20 minutes, 4 °C). The supernatant was loaded onto 1 mL cOmplete™ His-Tag purification resin (Roche) and calibrated three times with lysis buffer. The supernatant was incubated on the resin at 4 °C for two hours up to overnight. Non-specifically bound protein was removed by washing the resin three times with 10 mL wash buffer (50 mM Tris-HCl (pH 8), 300 mM NaCl, 0.1% Triton X-100) containing 5 mM imidazole and three times with 10 mL wash buffer containing 25 mM imidazole. Proteins bound to the resin were eluted with 10 mL elution buffer (50 mM Tris-HCl (pH 8), 300 mM NaCl, 0.1% Triton X-100) containing 500 mM imidazole. DTT and EDTA was added directly to a final concentration of 5 mM and 1 mM, respectively. The buffer was replaced with 2 × storage buffer (100 mM Tris-HCl (pH 8), 200 mM NaCl, 0.2% Triton X-100, 10 mM DTT, 2 mM EDTA, 1x cOmplete™ Protease-Inhibitor-Cocktail) using an Amicon 30,000 MWCO concentrator. Glycerol was added to a final concentration of 50% (v/v) and the proteins were stored at −20 °C.

### *In vitro* transcription assay and denaturing PAGE

Transcription reactions were performed for 60–90 minutes at 37–65 °C in a total reaction volume of 20 µL. The reaction mixtures contained 0.25–8 µM DNA template, 1–8 mM NTPs, 3.75–90 mM Mg(OAc)^2^, 1 U/µL RNase inhibitor and 0.25–2.5 µM T7 RNAP in 1 × transcription buffer (40 mM Tris-HCl (pH 8), 10 mM DTT, 2 mM spermidine). DNA templates were annealed by incubating the complementary oligonucleotides at 95 °C for 3 minutes followed by cooling to 4 °C (0.5 °C/s) in water. The templates contained a T7 promoter followed by the transcribed sequence. The sequence is reported 5′ to 3′ as the coding strand: 5′‑GCCTCCTATAATACGACTCACTATAGGGAGACCGTCAGCTGTGCCGTCGCGCAGCACGCGCCGCCGTGGACAGAGGACTGCAGAAAATCAACCTATCCTCCTTCAGGACCAACG-3′. The resulting 89 nt RNA transcript sequence was 5′-GGGAGACCGUCAGCUGUGCCGUCGCGCAGCACGCGCCGCCGUGGACAGAGGACUGCAGAAAAUCAACCUAUCCUCCUUCAGGACCAACG-3′. Reactions were initiated by the addition of T7 RNAP and transcripts were body labelled by incorporation of 16,65 nM 3000 Ci/mmol [α-^32^P]GTP in the reaction. Reactions were stopped by the addition of 100 µL PAGE stopping solution (90% formamide, 50 mM EDTA, 0.01% bromophenol blue, 0.01% xylene cyanol) and samples were denatured at 95 °C for 2 minutes. 0.8 µL of sample was analysed by electrophoresis on 10% denaturing PAGE gels and phosphorimaging.

### Analysis of heat inactivation

To determine residual activity after heat treatment, enzyme samples were preincubated for 5 minutes at temperatures between 25 °C and 70 °C in 1 × transcription buffer (40 mM Tris-HCl, pH 8.0, 2 mM spermidine, 10 mM DTT, 30 mM Mg(OAc)^2^). After pre-incubation, samples were assayed for residual activity at 37 °C using the *in vitro* transcription assay followed by analysis on denaturing PAGE.

### Assessment of dsRNA formation during transcription

To assess dsRNA formation, *in vitro* transcription was performed using the pGEM® Express Positive Control template (Promega, P2561). Transcription reactions (20 µL) contained 4 mM of each NTP, 1 × transcription buffer (40 mM Tris-HCl, pH 8.0, 2 mM spermidine, 10 mM DTT, 30 mM Mg(OAc)^2^), 1 U/µL RNase inhibitor, 0.05 g/L DNA template, and 1.5 µM T7 RNAP variant. Reactions were incubated at 37 °C or 48 °C for 90 minutes. Template DNA was then removed by treatment with DNase I (1 µL per 10 µL reaction volume) for 30 minutes at 37 °C. RNA was purified using the Zymo RNA Clean & Concentrator kit, and concentrations were determined spectrophotometrically using a NanoDrop instrument. For size and integrity analysis, 1 µL of a 1:5 dilution of each RNA sample was mixed with 5 µL RNA ScreenTape sample buffer, vortexed at 2,000 rpm for 1 minute (IKA MS3), denatured at 72 °C for 3 minutes, and immediately cooled on ice for 2 minutes. Samples were briefly centrifuged and analyzed on an Agilent 4150 TapeStation system according to the manufacturer’s instructions. For dsRNA detection, purified *in vitro* transcribed RNA was diluted in nuclease-free water to final amounts of 500 ng, 250 ng, or 100 ng in 10 µL and applied to a Nytran® SuPerCharge (SPC) nylon membrane using a Bio-Dot apparatus (Bio-Rad). Samples were allowed to bind to the membrane for 30 minutes, followed by two washing steps with 100 mL TBS-T per well. The membrane was then blocked for 1 hour at room temperature in 5% (w/v) non-fat dry milk in TBS-T. After blocking, the membrane was incubated overnight at 4 °C with the dsRNA-specific monoclonal J2 antibody (Scicons), diluted 1:5,000 in 1% (w/v) non-fat dry milk in TBS-T. The membrane was subsequently washed three times for 15–30 minutes each with TBS-T and incubated with the secondary antibody for 1 hour at room temperature in 1% (w/v) non-fat dry milk. Before detection, the membrane was washed again three times for 15–30 minutes each with TBS-T. Chemiluminescent signals were generated using freshly prepared peroxidase substrate (SuperSignal™ West Pico PLUS substrate, Thermo Scientific; mixed 1:1 Luminol/Enhancer and Stable Peroxidase Solution) and recorded with a ChemiDoc imaging system (Bio-Rad) (exposure time: 15 seconds).

## Supporting information

S1 Table Sequences of variants. (PDF)

S1 File Raw images. (PDF)
