# Supplementary material for: Engineering of T7 DNA-dependent RNA polymerase with activity at elevated temperature
Source: PLoS One. 2026 Jul 20;21(7):e0353775. doi: 10.1371/journal.pone.0353775 (PMC13384301; doi:10.1371/journal.pone.0353775)
Supplement: S1 Table — (PDF) [file pone.0353775.s001.pdf]

***S1 Table. Sequences of variants**. Mutation sites are marked in red.*

**T7 RNAP wt**	MNTINIAKND^{10} FSDIELAAIP^{20} FNTLADHYGE^{30}<br>RLAREQLALE^{40} HESYEMGEAR^{50} FRKMFERQLK^{60}<br>AGEVADNAAA^{70} KPLITTLLPK^{80} MIARINDWFE^{90}<br>EVKAKRGKRP^{100} TAFQFLQEIK^{110} PEAVAYITIK^{120}<br>TTLACLTSAD^{130} NTTVQAVASA^{140} IGRAIEDEAR^{150}<br>FGRIRDLEAK^{160} HFKKNVEEQL^{170} NKRVGHVYKK^{180}<br>AFMQVVEADM^{190} LSKGLLGGEA^{200} WSSWHKEDSI^{210}<br>HVGVRCIEML^{220} IESTGMVSLH^{230} RQNAGVVGQD^{240}<br>SETIELAPEY^{250} AEAIATRAGA^{260} LAGISPMFQP^{270}<br>CVVPPKPWTG^{280} ITGGGYWANG^{290} RRPLALVRTH^{300}<br>SKKALMRYED^{310} VYMPEVYKAI^{320} NIAQNTAWKI^{330}<br>NKKVLAVANV^{340} ITKWKHCPVE^{350} DIPAIEREEL^{360}<br>PMKPEDIDMN^{370} PEALTAWKRA^{380} AAAVYRKDKA^{390}<br>RKSRRISLEF^{400} MLEQANKFAN^{410} HKAIWFPYNM^{420}<br>DWRGRVYAVS^{430} MFNPQGNDMT^{440} KGLLTLAKGK^{450}<br>PIGKEGYYWL^{460} KIHGANCAGV^{470} DKVPFPERIK^{480}<br>FIEENHENIM^{490} ACAKSPLENT^{500} WWAEQDSPFC^{510}<br>FLAFCFEYAG^{520} VQHHGLSYNC^{530} SLPLAFDGSC^{540}<br>SGIQHFSAML^{550} RDEVGGRAVN^{560} LLPSETVQDI^{570}<br>YGIVAKKVNE^{580} ILQADAINGT^{590} DNEVVTVTDE^{600}<br>NTGEISEKVK^{610} LGTKALAGQW^{620} LAYGVTRSVT^{630}<br>KRSVMTLAYG^{640} SKEFGFRQQV^{650} LEDTIQPAID^{660}<br>SGKGLMFTQP^{670} NQAAGYMAKL^{680} IWESVSVTVV^{690}<br>AAVEAMNWLK^{700} SAAKLLAAEV^{710} KDKKTGEILR^{720}<br>KRCAVHWVTP^{730} DGFPVWQEYK^{740} KPIQTRLNLM^{750}<br>FLGQFRLQPT^{760} INTNKDSEID^{770} AHKQESGIAP^{780}<br>NFVHSQDGSH^{790} LRKTVVWAHE^{800} KYGIESFALI^{810}<br>HDSFGTIPAD^{820} AANLFKAVRE^{830} TMVDTYESCD^{840}<br>VLADFYDQFA^{850} DQLHESQLDK^{860} MPALPAKGNL^{870}<br>NLRDILESDF^{880} AFA
**PCD7**	MNTINIAKND^{10} FSDIELAAIP^{20} FNTLADHYGE^{30}<br>RLAREQLALE^{40} HESYEMGEAR^{50} FRKMFERQLK^{60}<br>AGEVADNAAA^{70} KPLITTLLPK^{80} MIARINDWFE^{90}<br>EVKAKRGKRP^{100} TAFQFLQEIK^{110} PEAVAYITIK^{120}<br>VILACLTSAD^{130} NTTVQAVASA^{140} IGRAIEDEAR^{150}<br>FGRIRDLEAK^{160} HFKKNVEEQL^{170} NKRVGHVYKK^{180}<br>AFMQVVEADM^{190} LSKGLLGGEA^{200} WSSWHKEDVI^{210}<br>HVGVRLIEML^{220} IESTGMVELH^{230} RQNAGVVGQD^{240}<br>SETIELAPEY^{250} AEAIATRAGA^{260} LAGISPMFQP^{270}<br>CVVPPKPWTG^{280} ITGGGYWANG^{290} RRPLALVRTH^{300}<br>SKKALMRYED^{310} VDMPEVYKAI^{320} NIAQNTAWKI^{330}<br>NKKVLEVANV^{340} ITKWKHCPVE^{350} DIPAIEREEL^{360}<br>PMKPEDIDMN^{370} PEALTAWKRA^{380} AAAVYRKDKA^{390}<br>RKSRRISLEF^{400} MLEQANKFAN^{410} HKAIWFPYNM^{420}<br>DWRGRVYAVS^{430} MFNPQGNDMT^{440} KGLLTLAKGK^{450}<br>PIGKEGYYWL^{460} KIHGANCAGV^{470} DKVPFDERIK^{480}

	WIEDNHENIM^{490} ACAKNPLENT^{500} WWAEQDSPFC^{510}<br>FLAFCFEYAG^{520} VQHHGLSYNC^{530} SLPLAFDGSC^{540}<br>SGIQHFSAML^{550} RDEVGGRAVN^{560} LLPSETVQDI^{570}<br>YQIVAKKVNE^{580} ILQADAINGT^{590} DNEVVTVTDE^{600}<br>NTGEISEKVK^{610} LGTKALAQQW^{620} LAYGVTRSVT^{630}<br>KRSVMTLAYG^{640} SKEFGFRQQV^{650} LEDTIQKAID^{660}<br>SGKGLMFTQP^{670} NQAAGYMAKL^{680} IWESVSQTVV^{690}<br>AAVEAMNWLK^{700} SAAKLLAAEV^{710} KDKKTGEILR^{720}<br>KRCAVHWVTP^{730} DGFPVWQEYK^{740} KPIQTRLNLM^{750}<br>FLGQFRLQPT^{760} INTNKDSEID^{770} AHKQESGIAP^{780}<br>NFVHSQDGSH^{790} LRKTVVWAHE^{800} KYGIESFALI^{810}<br>HDSFGTIPAD^{820} AENLFKAVRE^{830} TMVDTYESHD^{840}<br>VLADFYDQFA^{850} DQLHESQLDK^{860} MPPLPPKGNL^{870}<br>DLRDILESDF^{880} AFA
**PCD9**	MNTINIAKND^{10} FSDIELAAIP^{20} FNTLADHYGE^{30}<br>RLAREQLALE^{40} HESYEMGEER^{50} FRKMFERQLK^{60}<br>AGEVADNAAA^{70} KPLITTLLPK^{80} MIKRINDWFE^{90}<br>EVKAKRGKRP^{100} TAFQFLQEIK^{110} PEAIAYITIK^{120}<br>VILACLTSAD^{130} NTTVQAVASA^{140} IGRAIEDEAR^{150}<br>FGRIRDLEAK^{160} HFKKNVEEQL^{170} NKRVGHVYKK^{180}<br>AFMQVVEADM^{190} LSKGLLGGEA^{200} WSSWHKEDVI^{210}<br>HVGIRLIEML^{220} IESTGMVELH^{230} RQNAGVVGQD^{240}<br>SETIELTPEY^{250} AEAIAKRAGA^{260} LAGISPMFQP^{270}<br>CVVPPKPWTG^{280} ITGGGYWANG^{290} RRPLALVRTH^{300}<br>SKKALMRYED^{310} VDMPEVYKAI^{320} NIAQNTAWKI^{330}<br>NKKVLEVANV^{340} ITKWKHCPVE^{350} DIPAIEREEL^{360}<br>PMKPEDIDMN^{370} PEALTAWKRA^{380} AAAVYRKDKA^{390}<br>RKSRRISLEF^{400} MLEQANKFAN^{410} HEAIWFPYNM^{420}<br>DWRGRVYAVS^{430} MFNPQGNDMT^{440} KGLLTLAKGK^{450}<br>PIGEEGYYWL^{460} KIHGANCAGV^{470} DKVPFDERIK^{480}<br>WVEDNHENIM^{490} ACAKNPLENT^{500} WWAEQDSPFC^{510}<br>FLAFCFEYAG^{520} VQHHGLSYNC^{530} SLPLAFDGSC^{540}<br>SGIQHFSAML^{550} RDEVGGRAVN^{560} LLPSDTVQDI^{570}<br>YQIVAKKVNE^{580} LLKRDAINGT^{590} DNEVVTVTDE^{600}<br>NTGEITEKLK^{610} LGTKTLAQQW^{620} LAYGVTRSVT^{630}<br>KRSVMTLAYG^{640} SKEFGFRQQV^{650} LEDTIQKAID^{660}<br>NGKGLMFTQP^{670} NQAAGYMAKL^{680} IWEAVSQTVV^{690}<br>AAVEAMKWLK^{700} SAAKLLAAEV^{710} KDKKTGEVLR^{720}<br>PRCAVHWVTP^{730} DGFPVWQEYK^{740} KPIQTRLNLM^{750}<br>FLGQFRLQPT^{760} INTNKDSEID^{770} AHKQESGIAP^{780}<br>NFVHSQDGSH^{790} LRKTVVWAHE^{800} KYGIESFALI^{810}<br>HDSFGTIPAD^{820} AENLFKAVRE^{830} TFVDTYEKHD^{840}<br>VLADFYDQFA^{850} DQLHESQLDK^{860} MPPLPPKGNL^{870}<br>DLRDILESDF^{880} AFA
**P1H38D1**	MNTINIAKND^{10} FSDIEEAAIP^{20} FNTLADHYGE^{30}<br>RLAREQLALE^{40} HESYTMGEAR^{50} FRKMFERQLK^{60}<br>AGEVADNAAA^{70} KRLITTLLPK^{80} MIKRIKDWFE^{90}<br>EVKAKRGKRP^{100} TAFQFLQEIK^{110} PEAIAFITIK^{120}<br>TTLAMLLKPD^{130} NTTVTAVAIA^{140} IGRAIEDEAR^{150}

	FGRIRDQEAK^{160} HFKKNVEEQL^{170} NKRVGHVYKK^{180}<br>AFMQAVEADM^{190} LSKGLLGGEA^{200} WSSWHKEDSI^{210}<br>HVGLKLIEML^{220} IESTGMIELH^{230} RQHAGNPGRD^{240}<br>SETIELTPEW^{250} AEAIATRAGA^{260} LAGISPMFQP^{270}<br>CVVPPKPWTG^{280} ITGGGYWAPG^{290} RRPLALVRTH^{300}<br>SKKALMRYRD^{310} VDMPEVYKAI^{320} NIAQNTAWKI^{330}<br>NKKVLAVVNV^{340} ITKWKHVPVE^{350} DIPAIEREEL^{360}<br>PMKPEDIDMN^{370} PEALKAWKRK^{380} AAAVYRKDKA^{390}<br>RKSRRISLEF^{400} MLEQANKFAN^{410} HKAIWFPYQM^{420}<br>DWRGRVYAVS^{430} MFNPQGNDMT^{440} KGLLTLAKGK^{450}<br>PIGKEGYYWL^{460} KIHGANCAGV^{470} DKVPFEERIK^{480}<br>FIEENHENIM^{490} ACAKDPLENL^{500} WWAEQDSPFC^{510}<br>FLAFCFEYAG^{520} VQHHGLSYVC^{530} SLPLAFDGSC^{540}<br>SGIQHFSAML^{550} RDEVGGRAVN^{560} LLPSETVQDI^{570}<br>YGIVAKKVNE^{580} MLKADAINGT^{590} DNEVVTVTDE600<br>NTGEISEKVK^{610} LGTKALARQW^{620} LAFGVDRSVT^{630}<br>KRSVMTLAYG^{640} SKEFGFRQQV^{650} LEDTIKKAID^{660}<br>NGKGHMFTQP^{670} NQAAGYMAKL^{680} IWDSISVTVV^{690}<br>AAVEAMNWLK^{700} SAAKLLAAEV^{710} KDKKTGEILR^{720}<br>KRCAVHWVTP^{730} DGFPVWQEYK^{740} KPIQKRLDLM^{750}<br>FLGQFRLQPT^{760} INTNKDSEID^{770} AHKQESGIAP^{780}<br>NFVHSLDGSH^{790} LRKTVVHAHE^{800} KYGIESFALI^{810}<br>HDSFGTIPAD^{820} ADNLFKAVRE^{830} TFVDTYEKHD^{840}<br>VLADFYDQFA^{850} DQLHESQLDK^{860} MPALPPKGNL^{870}<br>NLRDILESDF^{880} AFA
**P1H38D3**	MNTINIAKND^{10} FSDIEEAAIP^{20} FNTLADHYGE^{30}<br>RLAREQLALE^{40} HEAYTMGEAR^{50} FRKMFERQLK^{60}<br>ARGEVADNAAA^{70} KRLITTLLPK^{80} MIKRIKDWFE^{90}<br>EVKAKRGKRP^{100} TAFQFLQEIK^{110} PEAIAFITIK^{120}<br>VTLAMLLKPD^{130} NTTVTAVAIA^{140} IGRAIEDEAR^{150}<br>FGRIRDQEAK^{160} HFKKNVEEQL^{170} NKRVGHHVYKK^{180}<br>AFMQAVEADM^{190} LSKGLLGGEA^{200} WSSWHKEDSI^{210}<br>HVGLKLIEML^{220} IESTGMIELH^{230} RQHAGDKKRD^{240}<br>SETIELTPEW^{250} AEAIATRAGA^{260} LAGISPMFQP^{270}<br>CVVPPKPWTG^{280} ITGGGYWAPG^{290} RRPLALVRTH^{300}<br>SKKALMRYRD^{310} VDMPEVYKAI^{320} NIAQNTAWKI^{330}<br>NKKVLAVVNV^{340} ITKWKHVPVE^{350} DIPAIEREEL^{360}<br>PMKPEDIDMN^{370} PEALKAWKRK^{380} AAAVYRKDKA^{390}<br>RKSRRISLEF^{400} MLEQANKFAN^{410} HKAIWFPYQM^{420}<br>DWRGRVYAVS^{430} MFNPQGNDMT^{440} KGLLTLAKGK^{450}<br>PIGKEGYYWL^{460} KIHGANCAGV^{470} DKVPFEERIK^{480}<br>FIEENHENIM^{490} ACAKDPLENL^{500} WWAEMDSPFC^{510}<br>FLAFCFEYAG^{520} VQHHGLSYVS^{530} SLPVAFDGSC^{540}<br>SGIQHFSAML^{550} RDEVGGRAVN^{560} LLPSETVQDI^{570}<br>YKIVAKKVNE^{580} MLKADAINGT^{590} DNEVVTVTDE^{600}<br>NTGEISEKVK^{610} LGTKALARQW^{620} LAFGVDRKVT^{630}<br>KRSVMTLAYG^{640} SKEFGFRQQV^{650} LEDTIKKAID^{660}<br>NGKGHMFTQP^{670} NQAAGYMAKL^{680} IWDAISVTVV^{690}<br>AAVEAMNWLK^{700} SAAKLLAAEV^{710} KDKKTGEILR^{720}

	KRCAVHWVTP^{730} DGFPVWQEYK^{740} KPIQKRLDLM^{750}<br>FLGQFRLQPT^{760} INTNKDSEID^{770} AHKQESGIAP^{780}<br>NFVHSMDGSH^{790} LRKTVVHAHE^{800} KYGIESFALI^{810}<br>HDSFGTIPAD^{820} ADNLFKAVRE^{830} TFVDTYEKHD^{840}<br>VLADFYDQFA^{850} DQLHESQLDK^{860} MPPLPPKGNL^{870}<br>NLQDILESDF^{880} AFA
**P1H38D5**	MNTINIAKND^{10} FSDIEEAAIP^{20} FNTLADHYGE^{30}<br>DLAREQLALE^{40} HEAYEMGEAR^{50} FRKMFERQLK^{60}<br>RGEVADNAAA^{70} KWLIATLLPK^{80} MIKRIKDWFE^{90}<br>EVKAKRGKRP^{100} TAFQFLQEIK^{110} PEAIAFITIK^{120}<br>VTLAMLLKKD^{130} NTTVTAVAIA^{140} IGRAIEDEAR^{150}<br>FGRIRDQEAK^{160} HFKKNVEEQL^{170} NKRVGHHYKK^{180}<br>AFMQAVEADM^{190} LSKGLLGGEA^{200} WSSWHKEDVI^{210}<br>HVGLKLIELL^{220} IESTGMIELH^{230} RQHAGDKTRD^{240}<br>RETIELTPEW^{250} AEWIATRAGA^{260} LAGISPMFQP^{270}<br>CVVPPKPWTG^{280} GITGGGYWAPG^{290} RRPLALVRTH^{300}<br>SKKALDRYRD^{310} VDMPEVYKAI^{320} NIAQNTAWKI^{330}<br>NKKVLAVVNV^{340} ITKWKHVPVE^{350} DIPAIEREEL^{360}<br>PMKPEDIDMN^{370} PEALKAWKRK^{380} AAAVYRKDKA^{390}<br>RKSRRISLEF^{400} MLEQANKFAN^{410} HKAIWFPYQL^{420}<br>DWRGRVYAVS^{430} MFNPQGNDMT^{440} KGLLTLAEGK^{450}<br>PIGKEGYYWL^{460} KIHGANCAGV^{470} DKVPFEERIK^{480}<br>FIEENHENIM^{490} ACAKNPLENL^{500} WWAEMDSPFC^{510}<br>FLAFCFEYAG^{520} VQHHGLSYVS^{530} SLPIAFDGSC^{540}<br>SGIQHFSAML^{550} RDEVGGRAVN^{560} LLPSETVQDI^{570}<br>YQIVAKKVNE^{580} KLKADAINGT^{590} DNEVETVVDE^{600}<br>DTGEISEKVK^{610} LGTKALARQW^{620} LAFGIDRKVT^{630}<br>KRSVMTLAYG^{640} SKEFGFRQQV^{650} LEDTIKKAID^{660}<br>NGKGHMFTQP^{670} NQAAGYMAKL^{680} IWDAIQVTVV^{690}<br>AAVEAMNWLK^{700} SAAKLLAAEV^{710} KDKKTGEILR^{720}<br>PRCAVHWVTP^{730} DGFPVWQEYY^{740} KPIQKRLDLM^{750}<br>FLGQFRLQPT^{760} INTNKDSEID^{770} AHKQESGIAP^{780}<br>NFVHSMDGSH^{790} LRKTVVHAHE^{800} KYGIESFALI^{810}<br>HDSFGTIPAD^{820} ADNLFKAVRE^{830} TFVDTYEKHD^{840}<br>VLADFYDQFA^{850} DQLHESQLDK^{860} MPPLPPKGNL^{870}<br>DLRDILESDF^{880} AFA
**P1H38D9**	MNTILIAKND^{10} FSDISHAAIP^{20} FNTLADHYGE^{30}<br>ELAREQLALE^{40} HEAYEMGEKR^{50} FLKMLERQRK^{60}<br>RGEEADSQVA^{70} KWLIATLLPK^{80} MIKRIKDWFE^{90}<br>EVKSKRGKRP^{100} TAFQFLQEID^{110} PEAIAFITIK^{120}<br>VVLAMLLKKD^{130} DTTVTAVAIA^{140} IGRAIEDEAR^{150}<br>FGRIRDQEAK^{160} HFKKHIEKNL^{170} NKRVGHHYKK^{180}<br>AYMQAVEADM^{190} LSKGLLGGEA^{200} WSSWHKEDVI^{210}<br>HVGLKLIELL^{220} IESTQLIEIH^{230} RQIAGDKTRD^{240}<br>RETIELTPEY^{250} AEWIAKRAGA^{260} LAGINPMFQP^{270}<br>CVVPPKPWTG^{280} ITGGGYWAPG^{290} RRPLALVRTH^{300}<br>SKKALDRYPD^{310} VDMPEVYKAI^{320} NIAQNTAWKI^{330}<br>NKKVLEVVNV^{340} IVKWKHVPVE^{350} DIPAIEREEL^{360}

	PMKPEDIDMN^{370} PEALKAWKRK^{380} AAAVYRKDKA^{390} RKSRRISLEF^{400} MLEQANKFAD^{410} YEAIWFPYQM^{420} DWRGRVYAVS^{430} MFNPQGNDMT^{440} KGLLTLAKGK^{450} PIGEEGYYWL^{460} MIHGANCAGV^{470} DKVPFEERIK^{480} WVEDNHENIM^{490} ACAKNPLDNT^{510} WWMEMDSPFC^{510} FLAFCFEYAG^{520} VQHHGLSYVC^{530} SLPIAFDGTC^{540} SGIQHFSAML^{550} RDEVGGRAVN^{560} LLPSDKPQDI^{570} YQIVAKKVNE^{580} KLKEDAKNGT^{590} DNEVETVTDK^{600} DTGEISEKTK^{610} LGTKTLARQW^{620} LAFGIDRKVT^{630} KRSVMTLAYG^{640} SKEFGFRQQV^{650} LEDIIKKAID^{660} NGKGHMFTQP^{670} NQAARYMAKL^{680} IWDAISQTVV^{690} AAVEAMDWLK^{700} AAAKLLAAEV^{710} KDKKTGEVLR^{720} PRLPVHWVTP^{730} DGFPVWQEYR^{740} KPIQTRLDLM^{750} FLGQFRLQPT^{760} INTNKDSEID^{770} AHKQESGIAP^{780} NFVHSMDAAH^{790} LRKTVVHAHE^{800} KYGIENFALI^{810} HDSFGTIPAD^{820} ADNLFKAVRE^{830} TFVEMYEKHD^{840} VLADFYEQFA^{850} DQLHESQLDK^{860} MPPLPPKGNL^{870} DLRDILESDF^{880} AFA
**M0 + G788A**	MNTINIAKND^{10} FSDIELAAIP^{20} FNTLADHYGE^{30} RLAREQLALE^{40} HESYEMGEAR^{50} FRKMFERQLK^{60} AGEVADNAAA^{70} KPLITTLLPK^{80} MIARINDWFE^{90} EVKAKRGKRP^{100} TAFQFLQEIK^{110} PEAVAYITIK^{120} TTLACLTSAD^{130} NTTVQAVASA^{140} IGRAIEDEAR^{150} FGRIRDLEAK^{160} HFKKNVEEQL^{170} NKRVGHVYKK^{180} AFMQVVEADM^{190} LSKGLLGGEA^{200} WSSWHKEDSI^{210} HVGVRCIEML^{220} IESTGMVSLH^{230} RQNAGVVGQD^{240} SETIELAPEY^{250} AEAIATRAGA^{260} LAGISPMFQP^{270} CVVPPKPWTG^{280} ITGGGYWANG^{290} RRPLALVRTH^{300} SKKALMRYED^{310} VYMPEVYKAI^{320} NIAQNTAWKI^{330} NKKVLAVANV^{340} ITKWKHCPVE^{350} DIPAIEREEL^{360} PMKPEDIDMN^{370} PEALTAWKRA^{380} AAAVYRKDKA^{390} RKSRRISLEF^{400} MLEQANKFAN^{410} HKAIWFPYNM^{420} DWRGRVYAVP^{430} MFTPQGNDMT^{440} KGLLTLAKGK^{450} PIGKEGYYWL^{460} KIHGANCAGV^{470} DKVPFPERIK^{480} FIEENHENIM^{490} ACAKSPLENT^{500} WWAEQDSPFC^{510} FLAFCFEYAG^{520} VQHHGLSYNC^{530} SLPLAFDGSC^{540} SGIQHFSAML^{550} RDEVGGRAVN^{560} LLPSETVQDI^{570} YGIVAKKVNE^{580} ILQADAINGT^{590} DNEVVTVTDE^{600} NTGEISEKVK^{610} LGTKALAGQW^{620} LAYGVTRSVT^{630} KRPVMTLAYG^{640} SKEFGFRQQV^{650} LEDTIQPAID^{660} SGKGLMFTQP^{670} NQAAGYMAKL^{680} IWESVSVTVV^{690} AAVEAMNWLK^{700} SAAKLLAAEV^{710} KDKKTGEILR^{720} KRCAVHWVTP^{730} DGFPVWQEYK^{740} KPIQTRLNLM^{750} FLGQFRLQPT^{760} INTNKDSEID^{770} AHKQESGIAP^{780} NFVHSQDASH^{790} LRKTVVWAHE^{800} KYGIESFALI^{810} HDSFGTIPAD^{820} AANLFKAVRE^{830} TMVDTYESCD^{840} VLADFYDQYA^{850} DQLHESQLDK^{860} MPALPAKGNL^{870} NLRDILESDF^{880} AFA
