# Supplementary material for: Engineering of T7 DNA-dependent RNA polymerase with activity at elevated temperature
Source: PLoS One. 2026 Jul 20;21(7):e0353775. doi: 10.1371/journal.pone.0353775 (PMC13384301; doi:10.1371/journal.pone.0353775)
Supplement: S1 File — (PDF) [file pone.0353775.s002.pdf]

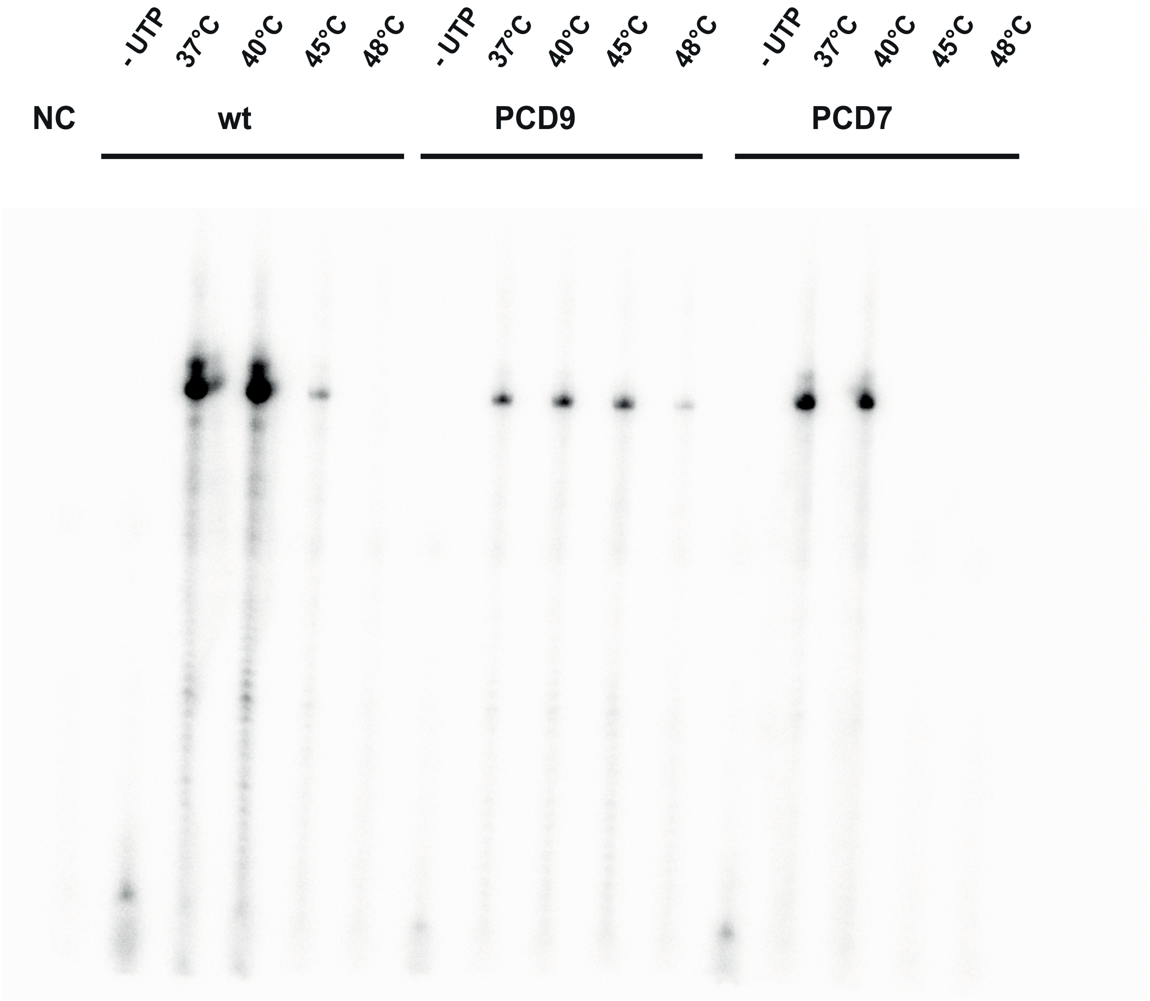

Raw image file Fig. 1D: Denaturing PAGE of a ^{32}P-based in vitro transcription assay comparing PCD7 and PCD9 with wild-type T7 RNAP (wt) at 37 °C, 40 °C, 45 °C, and 48 °C. Full-length transcription yields an 89 nt product. NC denotes a no-enzyme negative control, and −UTP indicates a reaction without UTP. Reactions were run for 90 min. The expected full-length product is 89 nt. 0.8 μL of sample was analysed by electrophoresis on 10 % denaturing PAGE gels and phosphorimaging using a Typhoon FLA 9500 Imager (GE Healthcare).

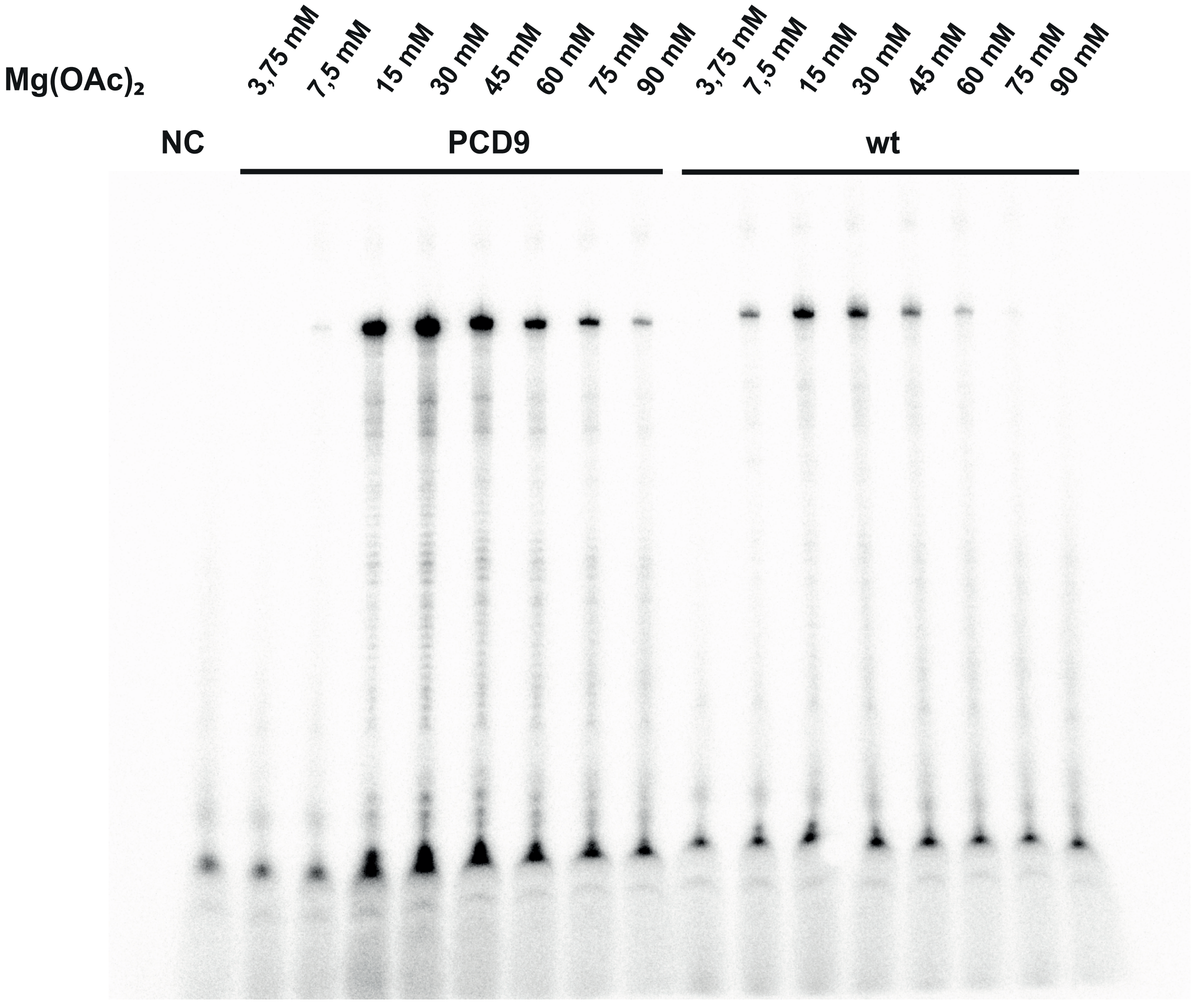

Raw image file Fig. 2A: Optimization of transcription conditions at 48 °C. Denaturing PAGE of a ^{32}P-based in vitro transcription assay testing the effects of $Mg(OAc)_2$ on RNA synthesis. Reactions were run for 90 min at 48 °C with PCD9 or wild-type T7 RNAP (wt) and included a no-enzyme control (NC). The expected full-length product is 89 nt. 0.8 µL of sample was analysed by electrophoresis on 10 % denaturing PAGE gels and phosphorimaging using a Typhoon FLA 9500 Imager (GE Healthcare).

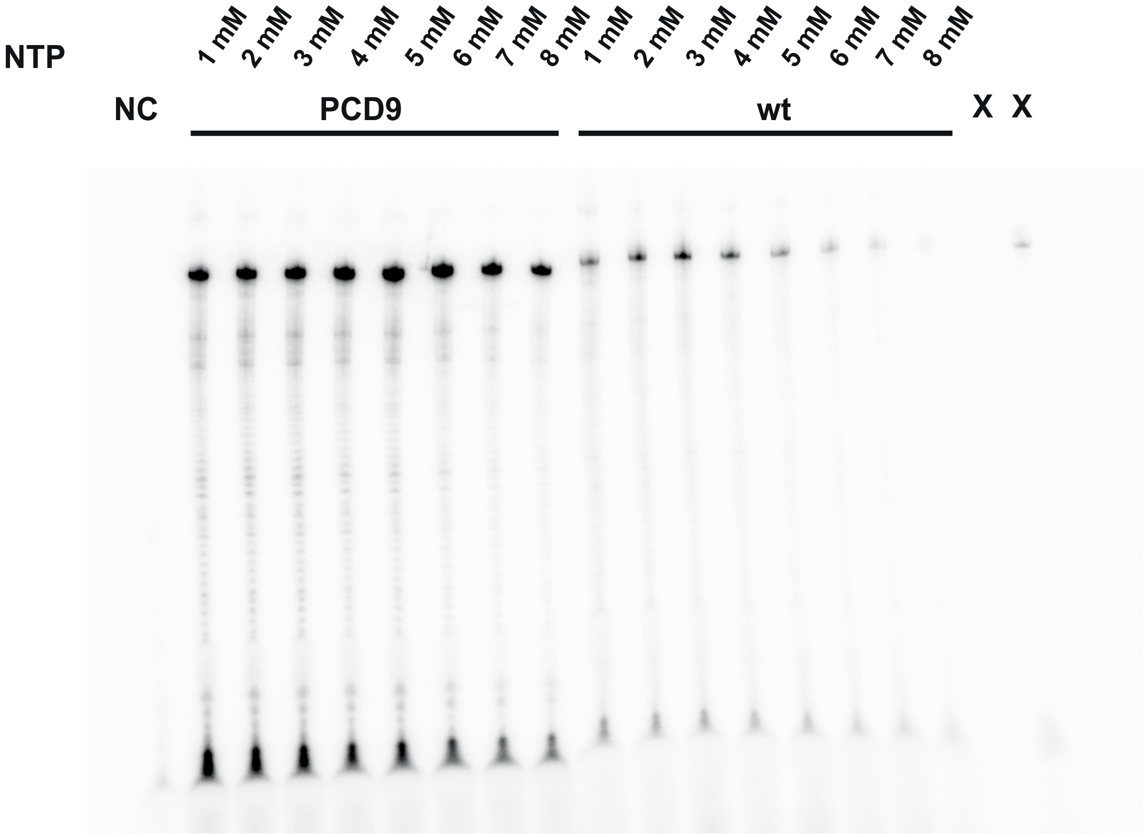

Raw image file Fig. 2C: Optimization of transcription conditions at 48 °C. Denaturing PAGE of a ^{32}P-based in vitro transcription assay testing the effects of NTP concentration on RNA synthesis. Reactions were run for 90 min at 48 °C with PCD9 or wild-type T7 RNAP (wt) and included a no-enzyme control (NC). The expected full-length product is 89 nt. 0.8 µL of sample was analysed by electrophoresis on 10 % denaturing PAGE gels and phosphorimaging using a Typhoon FLA 9500 Imager (GE Healthcare).

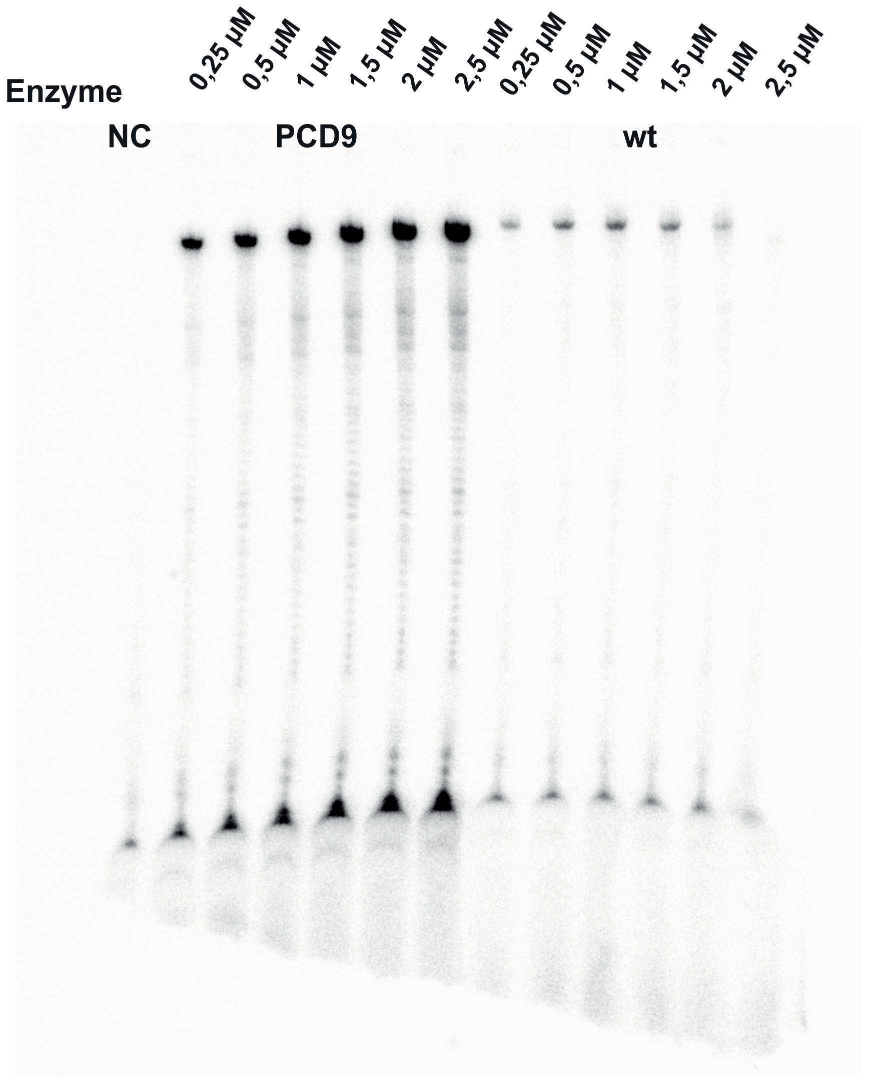

Raw image file Fig. 2E: Optimization of transcription conditions at 48 °C. Denaturing PAGE of a ^{32}P-based in vitro transcription assay testing the effects of enzyme concentration on RNA synthesis. Reactions were run for 90 min at 48 °C with PCD9 or wild-type T7 RNAP (wt) and included a no-enzyme control (NC). The expected full-length product is 89 nt. 0.8 µL of sample was analysed by electrophoresis on 10 % denaturing PAGE gels and phosphorimaging using a Typhoon FLA 9500 Imager (GE Healthcare).

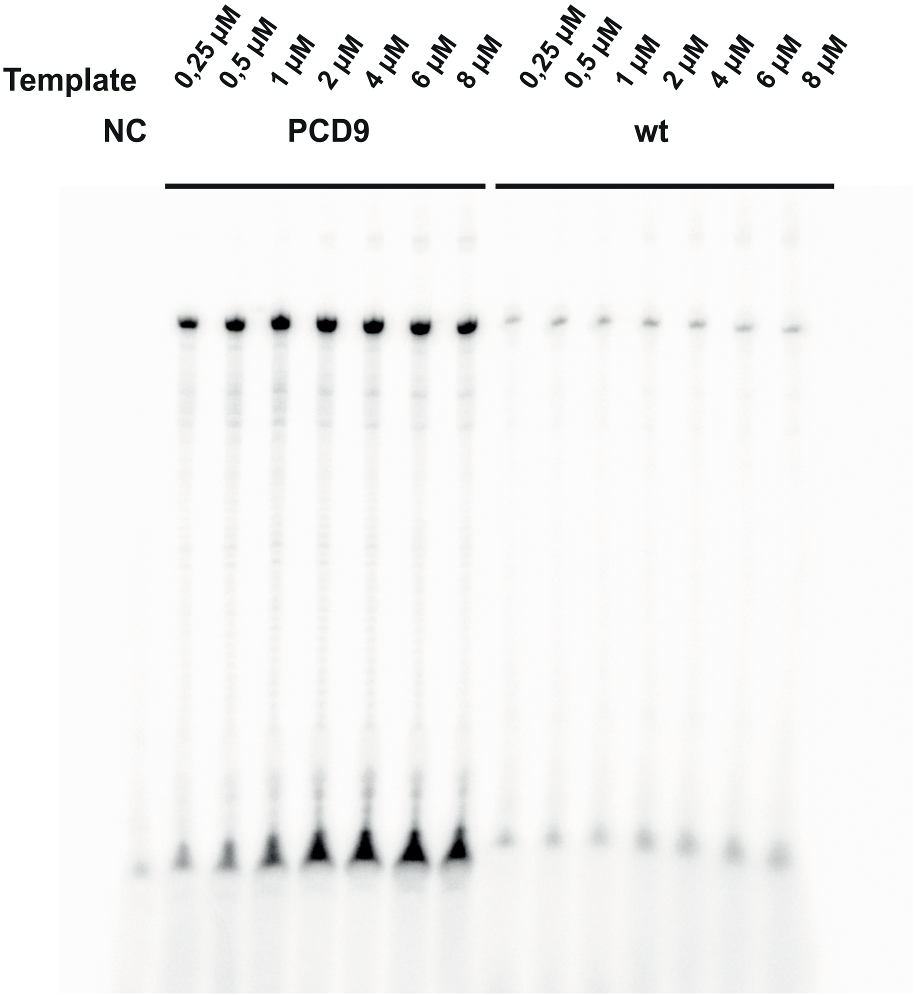

Raw image file Fig. 2F: Optimization of transcription conditions at 48 °C. Denaturing PAGE of a ^{32}P-based in vitro transcription assay testing the effects of template concentration on RNA synthesis. Reactions were run for 90 min at 48 °C with PCD9 or wild-type T7 RNAP (wt) and included a no-enzyme control (NC). The expected full-length product is 89 nt. 0.8 µL of sample was analysed by electrophoresis on 10 % denaturing PAGE gels and phosphorimaging using a Typhoon FLA 9500 Imager (GE Healthcare).

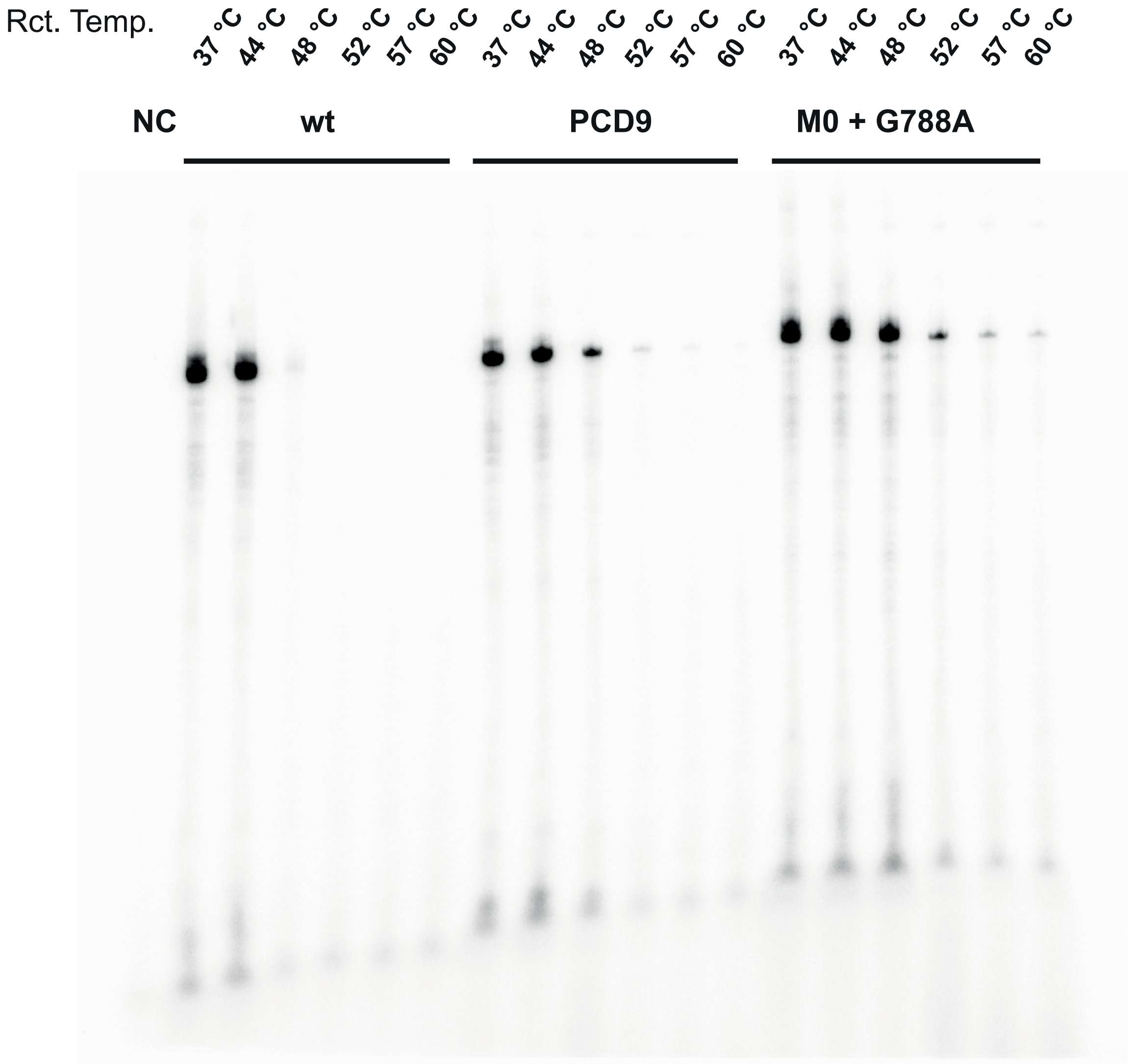

Raw image file Fig. 3A: Activity of T7 RNAP wild-type, PCD9 and M0 + G788A across temperatures from 37 to 60 °C. (A) Denaturing PAGE of a ^{32}P-based in vitro transcription assay with wt T7 RNAP (wt), PCD9, and M0 + G788A at temperatures of 37, 44, 48, 52, 57, and 60 °C. The expected full-length product is 89 nt. NC represents a negative control without enzyme. Reactions were run for 60 min. The expected full-length product is 89 nt. 0.8 µL of sample was analysed by electrophoresis on 10 % denaturing PAGE gels and phosphorimaging using a Typhoon FLA 9500 Imager (GE Healthcare).

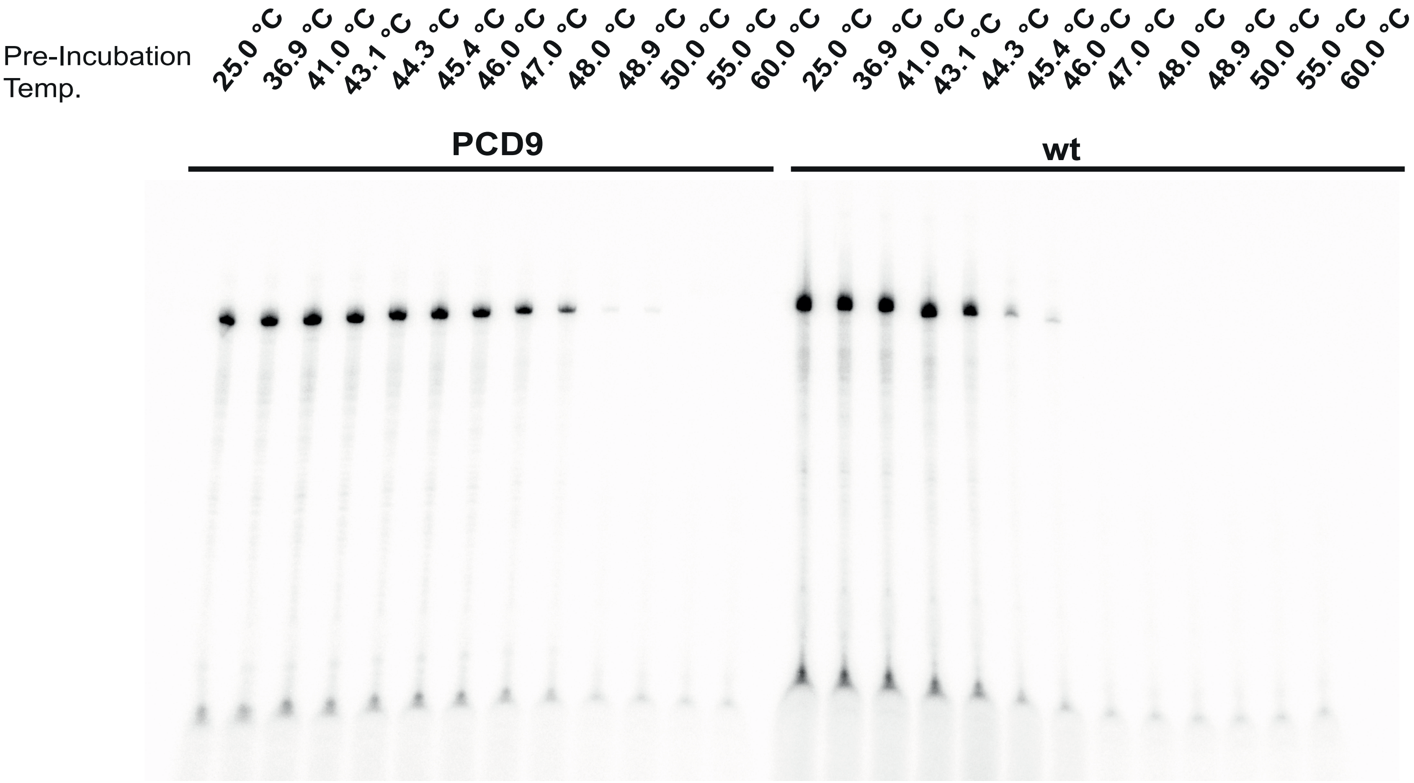

Raw image file Fig. 3C: Thermal inactivation of wt and PCD9. Enzyme variants were pre-incubated for 5 minutes at various temperatures (25.0, 36.9, 41.0, 43.1, 44.3, 45.4, 46.0, 47.0, 48.0, 48.9, 50.0, 55.0, and 60 °C) before the transcription reaction at 37 °C. Denaturing PAGE of a ^{32}P-based in vitro transcription assay is shown with full-length transcription yielding an 89 nt product. Reactions were run for 90 min. The expected full-length product is 89 nt. 0.8 µL of sample was analysed by electrophoresis on 10 % denaturing PAGE gels and phosphorimaging using a Typhoon FLA 9500 Imager (GE Healthcare).

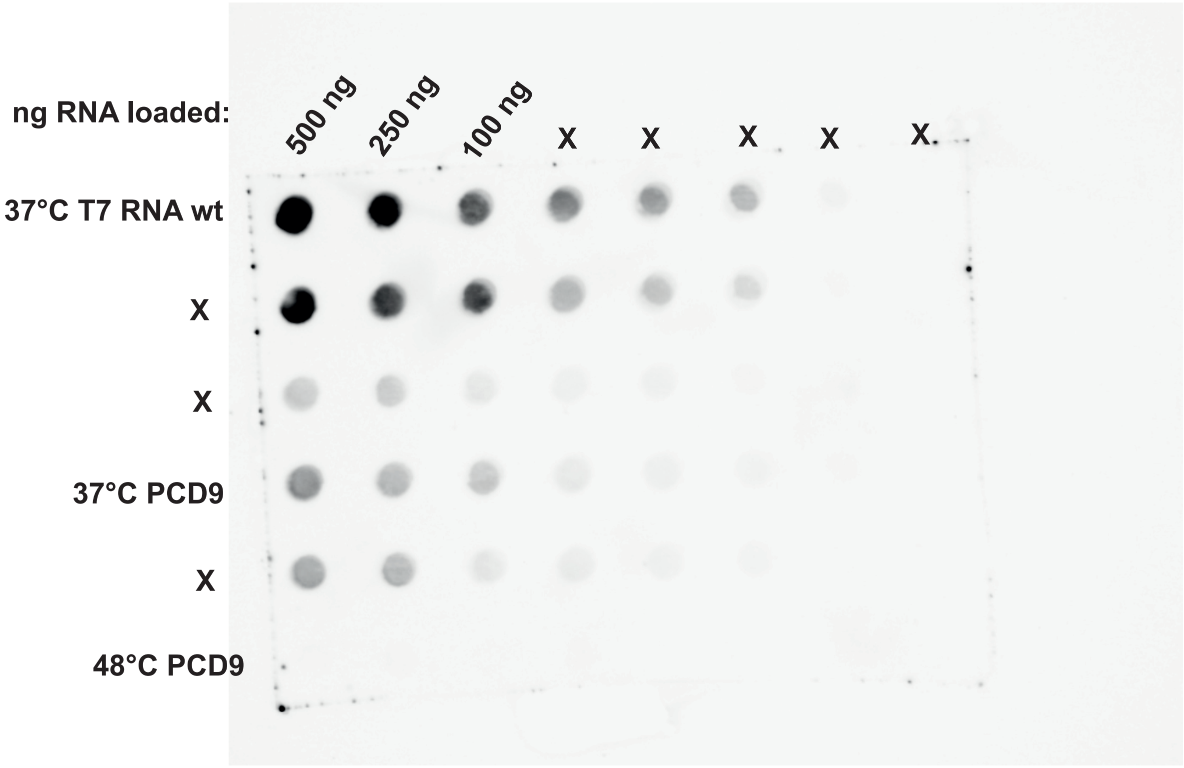

Raw image file Fig 4.C: dsRNA production by wild-type T7 RNAP and PCD9. Purified in vitro transcription reactions performed with wt T7 RNAP (wt) and PCD9 at 37 and 48 °C were analysed. Full-length transcription produced transcripts of 1,065 and 2,346 nt using the pGEM® Express Positive Control Template. dsRNA levels in the products were quantified by dot blot using 500 ng, 250 ng, and 100 ng per spot and detected with SCICONS's anti-dsRNA monoclonal antibody J2. Chemiluminescent signals were generated using freshly prepared peroxidase substrate (SuperSignal™ West Pico PLUS substrate, Thermo Scientific; mixed 1:1 Luminol/Enhancer and Stable Peroxidase Solution) and recorded with a ChemiDoc imaging system (Bio-Rad) (exposure time: 15 seconds).

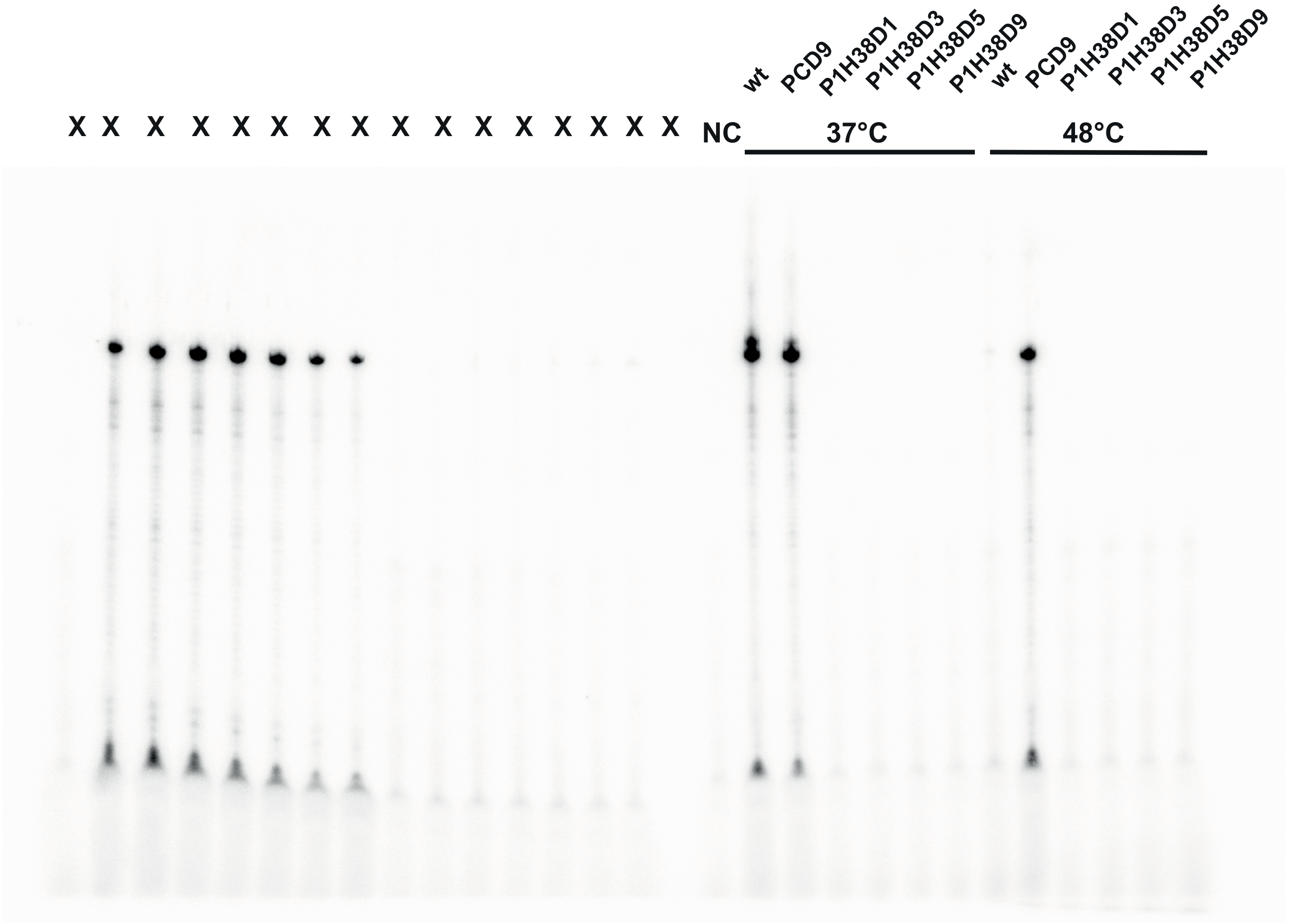

Raw image file Fig. 5: Activity of 1H38 PROSS designs 1, 3, 5, 9 at temperatures of 37 and 48 °C. Denaturating PAGE of ^{32}P-based in vitro transcription assay of the 1H38 PROSS designs 1, 3, 5, 9 at 37 and 48 °C in comparison to PCD9 and wt T7 RNAP (wt). Full length transcription yields in a 89 nt transcript. NC is a negative control without enzyme. Reactions were run for 90 min. 0.8 µL of sample was analysed by electrophoresis on 10 % denaturing PAGE gels and phosphorimaging using a Typhoon FLA 9500 Imager (GE Healthcare).
