# Supplementary material for: Engineering of T7 DNA-dependent RNA polymerase with activity at elevated temperature
Source: PLoS One. 2026 Jul 20;21(7):e0353775. doi: 10.1371/journal.pone.0353775 (PMC13384301; doi:10.1371/journal.pone.0353775)
Supplement: Attachment — Submitted filename: Response to Reviewers.docx [file pone.0353775.s004.docx]

Dear Editor and Reviewers,

We thank you for the careful evaluation of our manuscript and for the constructive comments. We appreciate the opportunity to revise our work. We have considered all editorial requirements and reviewer comments carefully and revised the manuscript accordingly. Below, we provide a point-by-point response and indicate how the manuscript has been changed.

Many thanks for your time and consideration.

**Responses to Journal Requirements**

**1. Compliance with PLOS ONE formatting, style, and file naming requirements**

We have checked the manuscript and associated files for compliance with PLOS ONE formatting, style, and file naming requirements. The figure files were prepared according to the journal requirements.

**2. Code sharing**

No author-generated code underpins the findings of this manuscript. The computational designs were generated using the publicly available PROSS web server, as described in the Methods section. Therefore, no code repository is applicable for this submission.

**3. Funding information**

We have checked and corrected the funding information to ensure consistency between the Funding Information and Financial Disclosure sections of the submission system. The correct funding information is:

“EU Horizon 2020 programme, NEWmRNA, Grant No. 965135.”

**4. Role of the funders statement**

We confirm the financial support from the EU Horizon 2020 programme, NEWmRNA, Grant No. 965135. As requested, we include the following Role of Funder statement in the cover letter:

“The funders had no role in study design, data collection and analysis, decision to publish, or preparation of the manuscript.”

**5. Removal of funding information from the manuscript**

All funding-related information has been removed from the manuscript file. Funding information will be provided only in the Funding Statement section of the online submission system, in accordance with PLOS ONE policy.

**6. Original uncropped gel and blot images**

We have ensured that all gel and blot figures comply with PLOS ONE image guidelines. The original uncropped and unadjusted images underlying all gel and blot data reported in the manuscript are provided in the Supporting Information file S2_Raw_images.

**7. Reviewer suggested citations**

We have reviewed the reviewer comments for recommendations to cite specific previously published works. No specific additional publications were recommended by the reviewers or required by the editor.

**Additional Correction Made During Revision:**

During revision, we also corrected an imprecise description of the structural input set used for the PROSS calculations. In the original manuscript, PDB 1MSW was referred to as a transition complex. Although the corresponding publication addresses the structural basis of the transition from initiation to elongation, the deposited structure represents an elongation complex. We have therefore revised the manuscript throughout to describe the input set as consisting of a promoter complex, an initiation complex, and two elongation complexes. We also adjusted the interpretation accordingly and now refer to the design as a multi-structure approach covering promoter recognition, initiation, and elongation-associated conformations, rather than four strictly discrete transcription states.

**Responses to Reviewer Comments**

**Reviewer 1:**

**Major Comments**
*A possible concern is that restricting the selection to only those mutations shared across all structures may lead to an overly conservative design strategy. This approach could potentially overlook mutations that are neutral with respect to other conformations, yet particularly beneficial for stabilizing or optimizing a specific conformation. The authors should discuss this limitation more explicitly and clarify why such conformation-selective mutations were not considered or prioritized.*

We agree with the reviewer that retaining only mutations shared across all structural inputs represents a conservative design strategy. This criterion was chosen to reduce the risk of introducing substitutions that stabilize one conformation while destabilizing another conformation required during the transcription cycle. However, we agree that this approach may exclude mutations that are neutral in most structural states but beneficial for a specific conformation. We have now added this limitation to the Discussion and clarified that conformation-selective mutations may provide an important additional design space for future engineering.

*Line 419: The authors state that, in the PROSS-based design, mutations at positions 537 and 812, which are important for T7RNAP activity, were excluded. However, to better preserve enzymatic function, would it not be more appropriate to fix not only these two positions, but also residues located within a few angstroms of the active site?*

We thank the reviewer for this important point. In the PROSS setup, residues 537 and 812 were excluded because they directly coordinate the catalytic divalent metal ions and are therefore essential for activity. In addition, after the PROSS calculations, all predicted substitutions were inspected for direct contacts with the DNA template or RNA transcript using PyMOL and PDIviz, and such positions were reverted to the wild type residue. We agree that fixing a broader shell around the active site could further reduce the risk of activity loss. However, a strict distance based exclusion of all residues within a few Å of the active site would also remove many positions in regions that undergo conformational rearrangements during transcription and might be overly restrictive for stability design. We have clarified this rationale in the Methods and added this point as a limitation.

*Line 172: The rationale for design selection requires further clarification. The authors appear to justify the choice on the basis that the number of mutations falls within the range reported for previous PROSS designs. However, it is not entirely clear whether this is an appropriate criterion, since the optimal number of introduced mutations is likely to vary substantially from one protein to another. In that sense, mutation counts reported for different proteins in prior studies may not be directly informative here. The authors should therefore provide a more specific justification for why PCD7 and PCD9 were selected over the other designs.*

We thank the reviewer for pointing this out. We agree that mutation numbers reported for other PROSS-designed proteins are only of limited value for selecting T7 RNAP variants, because the optimal mutational load is expected to be protein dependent. We have therefore revised the rationale for selecting PCD7 and PCD9. In the combined PROSS designs, designs 1 to 6 contained only 3 to 18 shared substitutions and were expected to provide a lower degree of stabilization. PCD7 was selected as a design with an intermediate mutational load, carrying 24 substitutions before filtering, whereas PCD9 represented the design with the highest mutational load, carrying 48 substitutions before filtering. This selection allowed us to test two different levels of the multi-structure design strategy. After removal of mutations predicted to contact nucleic acids, PCD7 and PCD9 contained 20 and 43 substitutions, respectively.

*Figure 4A: In the transcription reaction at 48°C using PCD9, a smeared RNA band is observed. This raises a potential practical concern, as transcription of incomplete or heterogeneous RNA products at elevated temperature could limit the utility of this variant for practical applications. The authors should comment on the possible cause of this band pattern and discuss whether it may represent a limitation for practical use.*

We agree that the broadened RNA profile observed for PCD9 at 48 °C is an important practical limitation. We have revised the Results and Discussion to state more clearly that, although PCD9 supports kilobase length transcription at 48 °C, the product distribution is more heterogeneous than at 37 °C. This may reflect increased pausing, premature termination, reduced elongation complex stability, or partial degradation during or after transcription. We also now state that this heterogeneous product profile could limit applications requiring highly homogeneous full-length RNA and may require further optimization of reaction conditions or downstream purification.

*Figure 4: The observation that PCD9 generates less dsRNA byproduct than WT at 37°C may simply be due to its lower transcriptional activity relative to WT. The authors should therefore consider showing the amount of dsRNA relative to the amount of ssRNA produced in each condition, so that dsRNA formation can be evaluated independently of differences in overall transcription yield.*

This is an important point. We would like to clarify that the dot blot assay was not performed using equal reaction volumes, but using defined amounts of purified RNA. Specifically, 500 ng, 250 ng, and 100 ng of purified RNA were loaded for each sample. Therefore, the lower dsRNA signal observed for PCD9 is not simply explained by lower total transcription yield, because the dsRNA signal was compared at equal total RNA mass. We have clarified this point in the Results and Methods. However, we agree that normalization to full length ssRNA specifically would provide an even more detailed analysis, particularly because the PCD9 reaction at 48 °C showed a broader product profile. We therefore now describe the result as reduced detectable dsRNA signal per amount of purified RNA and avoid overinterpreting it as a fully yield independent mechanistic reduction in dsRNA formation.

*Line 298: Please include a comparison with the M0 + G788A variant in the long-RNA transcription experiments and in the evaluation of dsRNA byproduct formation. Such a comparison would help clarify how the present design performs relative to a relevant benchmark mutant.*

We agree with the reviewer that including M0 + G788A in the long RNA transcription and dsRNA byproduct experiments would provide an additional useful benchmark. In the present study, these experiments were designed to characterize the newly identified PROSS-derived PCD9 variant under conditions where wt T7 RNAP is inactive. Therefore, the long transcript and dsRNA analyses focused on whether PCD9 itself can support kilobase-length RNA synthesis at 48 °C and whether this condition is associated with reduced detectable dsRNA signal.

M0 + G788A was included as a benchmark in the short transcript temperature series, which allows comparison of the temperature-dependent activity profiles of wt T7 RNAP, PCD9, and M0 + G788A under the same assay conditions. We have revised the manuscript to make the scope of the long transcript experiments clearer and now explicitly state that a direct comparison of PCD9 and M0 + G788A in kilobase-length RNA synthesis and dsRNA byproduct formation was not performed.

*The present results indicate that no thermostable variant outperforming the M0 + G788A mutant was obtained. This raises an important question as to whether this outcome reflects an inherent limitation of the PROSS-based design strategy in this system. The authors should discuss this point more explicitly. In particular, it would be valuable to consider what additional design principles may be required to obtain variants that surpass those generated by existing approaches. A deeper discussion would help clarify both the limitations of the current approach and the future direction for engineering superior variants.*

We agree with the reviewer that this point requires a more explicit discussion. We have revised the Discussion to clarify that PCD9 improves thermotolerance relative to wt T7 RNAP, but does not reach the high temperature activity of M0 + G788A in the short transcript assay. We do not interpret this as an inherent limitation of PROSS. Rather, it likely reflects the difference between a stability focused design starting from wt T7 RNAP and a benchmark variant that already combines previously validated thermostabilizing mutations in an activity compatible background. We now discuss that future engineering strategies may need to use established thermotolerant backgrounds such as M0 + G788A as starting points, introduce PROSS predicted substitutions in smaller spatially or functionally grouped sets, and use partial revertant or recombination approaches to identify which PCD9 substitutions are beneficial, neutral, or detrimental.

**Minor Comments**

*1. In the Results section, Figure 1D is described before Figure 1C. Please revise the order of the figure labels so that they follow the order in which they are discussed in the text.*

We have revised the order of the figure labels.

*2. In Figure 1C, the active site in the structure should be clearly highlighted, for example by using distinct colors or other visual markers.*

We have revised Figure 1D (former Figure 1C) to highlight the active site using a distinct visual marker.
 *3. The Results subsection associated with Figure 1 lacks a clear concluding statement. Please add a brief statement summarizing the main takeaway from this result.*

We have added a concluding statement to the Results subsection associated with Figure 1 to summarize the main takeaway. The revised text now states that the multi-structure PROSS workflow identified PCD9, but not PCD7, as a T7 RNAP variant with improved transcriptional activity at elevated temperature relative to wt T7 RNAP.

*4. For the condition optimization shown in Figure 2, please specify the temperature at which these experiments were performed near the beginning of the corresponding Results subsection.*

We have revised the beginning of the Results subsection associated with Figure 2 to specify that the buffer optimization experiments were performed at 48 °C.

*5. Figure 3A and 3B, as well as Figure 4A-C, are not cited in the Results text. Please ensure that all panels are explicitly referenced and appropriately discussed.*

We have revised the Results section to explicitly cite and discuss all figure panels.

**Reviewer 2:**

*1. The authors did refer to some of the modifications being researched or used in prior studies that talks about how the change in that particular amino acid (or a bunch of amino acids) has changed the functionality of T7 RNA polymerase. I would highly recommend talking about the most important changes in the amino acid configuration of PCD9 and how mechanistically it might have changed the thermostability. I understand that random mutations, sometimes might bring us to the solutions we want, but understanding the core of the solution and linking that to the actual reasons is what matters. For example, some of the mutations resembles with T3 RNA polymerase, another family of viral polymerase. Some of the other ones are in a loop, that might be more tolerable to such mutations (in other words can be redundant).*

We thank the reviewer for this helpful suggestion. We agree that a more detailed mechanistic interpretation of the PCD9 mutation set would be valuable. However, because PCD9 contains 43 substitutions and individual single mutants or revertants were not analyzed, assigning specific mechanistic roles to each substitution would be highly speculative. The manuscript already discusses substitutions that overlap with previously reported thermostable variants, including S684A and positions corresponding to A83, K454, A584, and M832 in variants reported by Ong et al. To further address the reviewer’s point, we have added a cautious discussion of substitutions located in loop or surface exposed regions. Specifically, we now note that PCD9 substitutions such as I718V and K721P may be more readily tolerated because they are less likely to directly interfere with catalysis or nucleic acid binding. Such substitutions could influence local flexibility or stabilize structural elements, but they may also be functionally neutral in the PCD9 background. We also agree that comparison with homologous phage RNA polymerases, such as T3 RNAP, could provide additional insight into whether selected substitutions correspond to naturally tolerated amino acid identities in related enzymes. However, such an analysis was not performed in the present study, and we therefore avoid assigning individual PCD9 mutations to a T3 RNAP-like sequence context.

*2. You noted that PCD9 produced visibly weaker dsRNA signals than the wild-type enzyme even at 37 °C, suggesting that the reduction in dsRNA might not rely solely on temperature, but could involve altered reaction kinetics or abortive transcription rates. Can you provide any kinetic data (e.g., Km, Vmax) to support this hypothesis and clarify how the mutations affect baseline activity?*

We agree that kinetic measurements would be required to determine whether the reduced dsRNA signal of PCD9 is linked to altered Km, Vmax, initiation efficiency, elongation rate, or abortive transcription. These kinetic parameters were not determined in the present study. We have therefore revised the manuscript to avoid implying a mechanistic conclusion. The text now states that altered reaction kinetics or abortive transcription may contribute to the observed reduction in dsRNA signal, but that this remains to be tested.

*3. The thermal inactivation assay involved pre-incubating the enzymes in a buffer devoid of NTP substrates and DNA template. Because ligand binding often stabilizes proteins, how might the presence of these active transcription components shift the temperature tolerance, and would testing in their presence provide a more accurate reflection of operational stability?*

We agree that pre-incubation in the absence of NTP substrates and DNA template may underestimate operational stability during active transcription, because binding of template DNA, nascent RNA, NTPs, and divalent metal ions could stabilize the transcription complex and shift the onset of activity loss to higher temperatures. This limitation was already noted in the Results section, and we have revised the wording to make the distinction between heat-induced inactivation under non-transcribing conditions and operational stability during active RNA synthesis clearer.

*4. When transcribing the kilobase-length template at 48 °C, the TapeStation electropherogram showed a broadened profile with shorter RNA species, which you attribute to potential polymerase pausing and premature termination. Have the authors checked for any sequence specificity of this pause/termination? How significantly does this truncation affect the overall quantitative yield of the full-length therapeutic-grade transcript? Can it be an effect of presence of trace amounts of salts in the purified RNA?*

We thank the reviewer for this important comment. We did not determine whether the shorter RNA species observed for PCD9 at 48 °C arise from sequence-specific pausing or termination. We have therefore revised the manuscript to avoid assigning the broadened TapeStation profile to a single cause. The revised text now states that the presence of shorter RNA species may reflect reduced stability of the elongation complex at 48 °C, increased polymerase pausing or premature termination, or partial RNA degradation during or after transcription.

Although TapeStation analysis provides an overview of the RNA size distribution, it was not sufficient to precisely quantify the full-length fraction in this case. The PCD9 sample at 48 °C showed a broad electropherogram profile with partially overlapping RNA species, and the template itself generates two expected transcript products of 1,065 nt and 2,346 nt. Therefore, reliable assignment and integration of full-length and truncated fractions was not possible from the present data. We have clarified this limitation in the revised manuscript. Future analyses using orthogonal higher resolution methods, such as analytical RNA chromatography, would be required to quantify the full-length fraction more precisely.

Regarding residual salts, the RNA was purified before TapeStation analysis, making residual salts unlikely to be the primary cause of the broadened profile. However, minor effects from trace contaminants cannot be fully excluded.

*5. No sequence information for the RNA (both short and long) is available. Addgene plasmid number, or sequence information in another format is required. (Ignore it if already submitted in a publicly accessible database that I have missed)*

We have added the sequence of the short RNA transcript to the Methods section. The long transcript assay was performed using the commercial pGEM® Express Positive Control Template from Promega and not an Addgene plasmid. Therefore, no Addgene plasmid number is applicable. To make the template source unambiguous, we have added further identifying information, including the supplier and product number, to the Methods section.

*6. It would be interesting to see all the modified protein in a native protein gel electrophoresis and how it compares to wild-type in folding.*

We thank the reviewer for this helpful suggestion. We agree that native PAGE would be a useful and accessible additional experiment to compare the purified T7 RNAP variants with wt T7 RNAP and to assess potential differences in electrophoretic behavior, apparent folding behavior, and aggregation state. In the present study, we focused on functional characterization through soluble expression, purification, and transcription activity assays. We have therefore not included native PAGE in the revised manuscript, but have added this point to the Discussion as a useful future experiment. Such analysis, ideally combined with complementary biophysical methods, could help further evaluate whether the introduced mutations affect folding behavior and thermal stability.
