# Supplementary material for: Engineering of T7 DNA-dependent RNA polymerase with activity at elevated temperature
Source: PLoS One. 2026 Jul 20;21(7):e0353775. doi: 10.1371/journal.pone.0353775 (PMC13384301; doi:10.1371/journal.pone.0353775)
Supplement: Attachment — Submitted filename: Second Response to Reviewers.docx [file pone.0353775.s005.docx]

Dear Editor and Reviewers,

We thank you for the careful evaluation of our revised manuscript in this second round of review. We appreciate the positive assessment of our revision and the opportunity to address the remaining minor points. We have carefully considered the editorial requirements and reviewer comments and revised the manuscript accordingly. Below, we provide a point-by-point response and indicate how the manuscript has been changed.

Many thanks for your time and consideration.

**Responses to Journal Requirements**

Comment:
*If the reviewer comments include a recommendation to cite specific previously published works, please review and evaluate these publications to determine whether they are relevant and should be cited. There is no requirement to cite these works unless the editor has indicated otherwise.*

Response:
The reviewer comments did not include any recommendations to cite specific previously published works. Therefore, no additional references were added in response to this requirement.

Comment:
*Please review your reference list to ensure that it is complete and correct. If you have cited papers that have been retracted, please include the rationale for doing so in the manuscript text, or remove these references and replace them with relevant current references.*

Response:
We have reviewed the reference list for completeness and correctness and made minor formatting corrections. No retracted articles are cited in the manuscript.

**Responses to Reviewer Comments**

**Reviewer 1:**

Comment:
*The revised manuscript is acceptable after a minor correction. Please confirm whether “Figure 1D” on line 190 should be “Figure 1C,” and whether “Figure 1C” on line 205 should be “Figure 1D.” Once this issue is addressed, I recommend acceptance.*

Response:
We thank the reviewer for carefully reviewing our manuscript for a second round and for pointing this out. We have checked the figure references and corrected them accordingly. The reference on line 190 now refers to Figure 1C, and the reference on line 205 now refers to Figure 1D.

**Reviewer 2:**

Comment:
*The authors have provided satisfactory responses to all comments to the best of their ability.*

Response:
We thank the reviewer for carefully reviewing our manuscript for a second round and for the positive assessment of our revised manuscript.
