# Supplementary material for: Engineering of T7 DNA-dependent RNA polymerase with activity at elevated temperature
Source: PLoS One. 2026 Jul 20;21(7):e0353775. doi: 10.1371/journal.pone.0353775 (PMC13384301; doi:10.1371/journal.pone.0353775)
Supplement: Peer review history

# Peer review: Decision Letter 0

**DOI:** 10.1371/journal.pone.0353775.r001

**Authors:** Jian Xu

12 May 2026

PONE-D-26-10563

Engineering of T7 DNA-dependent RNA polymerase with activity at elevated temperature

PLOS One

Dear Dr. Marx,

Thank you for submitting your manuscript to PLOS ONE. After careful consideration, we feel that it has merit but does not fully meet PLOS ONE’s publication criteria as it currently stands. Therefore, we invite you to submit a revised version of the manuscript that addresses the points raised during the review process.

As you can see the comments, both two reviewers raised some major and minor issues of your study, which need significant modifications in this round of the revision.

Please submit your revised manuscript by Jun 26 2026 11:59PM. If you will need more time than this to complete your revisions, please reply to this message or contact the journal office at plosone@plos.org. When you're ready to submit your revision, log on to https://www.editorialmanager.com/pone/ and select the 'Submissions Needing Revision' folder to locate your manuscript file.

Please include the following items when submitting your revised manuscript:

- A letter that responds to each point raised by the academic editor and reviewer(s). You should upload this letter as a separate file labeled 'Response to Reviewers'.
- A marked-up copy of your manuscript that highlights changes made to the original version. You should upload this as a separate file labeled 'Revised Manuscript with Track Changes'.
- An unmarked version of your revised paper without tracked changes. You should upload this as a separate file labeled 'Manuscript'.

If you would like to make changes to your financial disclosure, please include your updated statement in your cover letter. Guidelines for resubmitting your figure files are available below the reviewer comments at the end of this letter.

If applicable, we recommend that you deposit your laboratory protocols in protocols.io to enhance the reproducibility of your results. Protocols.io assigns your protocol its own identifier (DOI) so that it can be cited independently in the future. For instructions see: https://journals.plos.org/plosone/s/submission-guidelines#loc-laboratory-protocols. Additionally, PLOS ONE offers an option for publishing peer-reviewed Lab Protocol articles, which describe protocols hosted on protocols.io. Read more information on sharing protocols at https://plos.org/protocols?utm_medium=editorial-email&utm_source=authorletters&utm_campaign=protocols.

As the corresponding author, your ORCID iD is verified in the submission system and will appear in the published article. PLOS supports the use of ORCID, and we encourage all coauthors to register for an ORCID iD and use it as well. Please encourage your coauthors to verify their ORCID iD within the submission system before final acceptance, as unverified ORCID iDs will not appear in the published article. *Only*  the individual author can complete the verification step; PLOS staff *cannot*  verify ORCID iDs on behalf of authors.

We look forward to receiving your revised manuscript.

Kind regards,

Jian Xu, Ph.D.

Academic Editor

PLOS One

**Journal Requirements:**

When submitting your revision, we need you to address these additional requirements.

1. Please ensure that your manuscript meets PLOS ONE's style requirements, including those for file naming. The PLOS ONE style templates can be found at

https://journals.plos.org/plosone/s/file?id=wjVg/PLOSOne_formatting_sample_main_body.pdf and

https://journals.plos.org/plosone/s/file?id=ba62/PLOSOne_formatting_sample_title_authors_affiliations.pdf

2. Please note that PLOS One has specific guidelines on code sharing for submissions in which author-generated code underpins the findings in the manuscript. In these cases, we expect all author-generated code to be made available without restrictions upon publication of the work. Please review our guidelines at https://journals.plos.org/plosone/s/materials-and-software-sharing#loc-sharing-code and ensure that your code is shared in a way that follows best practice and facilitates reproducibility and reuse.

3. We note that the grant information you provided in the ‘Funding Information’ and ‘Financial Disclosure’ sections do not match.

When you resubmit, please ensure that you provide the correct grant numbers for the awards you received for your study in the ‘Funding Information’ section.

4. Thank you for stating the following financial disclosure:

“This research was funded by the EU Horizon 2020 programme (NEWmRNA, Grant No. 965135).”

Please state what role the funders took in the study.  If the funders had no role, please state: "The funders had no role in study design, data collection and analysis, decision to publish, or preparation of the manuscript."

If this statement is not correct you must amend it as needed.

Please include this amended Role of Funder statement in your cover letter; we will change the online submission form on your behalf.

5. Please note that funding information should not appear in any section or other areas of your manuscript. We will only publish funding information present in the Funding Statement section of the online submission form. Please remove any funding-related text from the manuscript.

6. PLOS ONE now requires that authors provide the original uncropped and unadjusted images underlying all blot or gel results reported in a submission’s figures or Supporting Information files. This policy and the journal’s other requirements for blot/gel reporting and figure preparation are described in detail at https://journals.plos.org/plosone/s/figures#loc-blot-and-gel-reporting-requirements and https://journals.plos.org/plosone/s/figures#loc-preparing-figures-from-image-files. When you submit your revised manuscript, please ensure that your figures adhere fully to these guidelines and provide the original underlying images for all blot or gel data reported in your submission. See the following link for instructions on providing the original image data: https://journals.plos.org/plosone/s/figures#loc-original-images-for-blots-and-gels.

In your cover letter, please note whether your blot/gel image data are in Supporting Information or posted at a public data repository, provide the repository URL if relevant, and provide specific details as to which raw blot/gel images, if any, are not available. Email us at plosone@plos.org if you have any questions.

7. If the reviewer comments include a recommendation to cite specific previously published works, please review and evaluate these publications to determine whether they are relevant and should be cited. There is no requirement to cite these works unless the editor has indicated otherwise.

[Note: HTML markup is below. Please do not edit.]

Reviewers' comments:

Reviewer's Responses to Questions

**Comments to the Author**

1. Is the manuscript technically sound, and do the data support the conclusions?

The manuscript must describe a technically sound piece of scientific research with data that supports the conclusions. Experiments must have been conducted rigorously, with appropriate controls, replication, and sample sizes. The conclusions must be drawn appropriately based on the data presented.

Reviewer #1: Partly

Reviewer #2: Partly

**********

2. Has the statistical analysis been performed appropriately and rigorously?

Reviewer #1: N/A

Reviewer #2: Yes

**********

3. Have the authors made all data underlying the findings in their manuscript fully available?

The PLOS Data policy requires authors to make all data underlying the findings described in their manuscript fully available without restriction, with rare exception (please refer to the Data Availability Statement in the manuscript PDF file). The data should be provided as part of the manuscript or its supporting information, or deposited to a public repository. For example, in addition to summary statistics, the data points behind means, medians and variance measures should be available. If there are restrictions on publicly sharing data—e.g. participant privacy or use of data from a third party—those must be specified.

Reviewer #1: Yes

Reviewer #2: No

**********

4. Is the manuscript presented in an intelligible fashion and written in standard English?

PLOS ONE does not copyedit accepted manuscripts, so the language in submitted articles must be clear, correct, and unambiguous. Any typographical or grammatical errors should be corrected at revision, so please note any specific errors here.

Reviewer #1: No

Reviewer #2: Yes

**********

5. Review Comments to the Author

Please use the space provided to explain your answers to the questions above. You may also include additional comments for the author, including concerns about dual publication, research ethics, or publication ethics. (Please upload your review as an attachment if it exceeds 20,000 characters)

Reviewer #1: The authors examined whether taking multiple conformational states of an enzyme into account is important for designing mutations that improve protein stability without compromising function. Using multiple structural datasets of T7 RNA polymerase and PROSS, they identified shared candidate mutations and generated variants with enhanced thermostability while maintaining catalytic activity. The study addresses an important question in protein engineering and explores a potentially useful framework for improving protein stability while attempting to preserve function. However, several issues need to be addressed before the manuscript can be considered for publication.

Major Comments

A possible concern is that restricting the selection to only those mutations shared across all structures may lead to an overly conservative design strategy. This approach could potentially overlook mutations that are neutral with respect to other conformations, yet particularly beneficial for stabilizing or optimizing a specific conformation. The authors should discuss this limitation more explicitly and clarify why such conformation-selective mutations were not considered or prioritized.

Line 419: The authors state that, in the PROSS-based design, mutations at positions 537 and 812, which are important for T7RNAP activity, were excluded. However, to better preserve enzymatic function, would it not be more appropriate to fix not only these two positions, but also residues located within a few angstroms of the active site?

Line 172: The rationale for design selection requires further clarification. The authors appear to justify the choice on the basis that the number of mutations falls within the range reported for previous PROSS designs. However, it is not entirely clear whether this is an appropriate criterion, since the optimal number of introduced mutations is likely to vary substantially from one protein to another. In that sense, mutation counts reported for different proteins in prior studies may not be directly informative here. The authors should therefore provide a more specific justification for why PCD7 and PCD9 were selected over the other designs.

Figure 4A: In the transcription reaction at 48°C using PCD9, a smeared RNA band is observed. This raises a potential practical concern, as transcription of incomplete or heterogeneous RNA products at elevated temperature could limit the utility of this variant for practical applications. The authors should comment on the possible cause of this band pattern and discuss whether it may represent a limitation for practical use.

Figure 4: The observation that PCD9 generates less dsRNA byproduct than WT at 37°C may simply be due to its lower transcriptional activity relative to WT. The authors should therefore consider showing the amount of dsRNA relative to the amount of ssRNA produced in each condition, so that dsRNA formation can be evaluated independently of differences in overall transcription yield.

Line 298: Please include a comparison with the M0 + G788A variant in the long-RNA transcription experiments and in the evaluation of dsRNA byproduct formation. Such a comparison would help clarify how the present design performs relative to a relevant benchmark mutant.

The present results indicate that no thermostable variant outperforming the M0 + G788A mutant was obtained. This raises an important question as to whether this outcome reflects an inherent limitation of the PROSS-based design strategy in this system. The authors should discuss this point more explicitly. In particular, it would be valuable to consider what additional design principles may be required to obtain variants that surpass those generated by existing approaches. A deeper discussion would help clarify both the limitations of the current approach and the future direction for engineering superior variants.

Minor Comments

1. In the Results section, Figure 1D is described before Figure 1C. Please revise the order of the figure labels so that they follow the order in which they are discussed in the text.

2. In Figure 1C, the active site in the structure should be clearly highlighted, for example by using distinct colors or other visual markers.

3. The Results subsection associated with Figure 1 lacks a clear concluding statement. Please add a brief statement summarizing the main takeaway from this result.

4. For the condition optimization shown in Figure 2, please specify the temperature at which these experiments were performed near the beginning of the corresponding Results subsection.

5. Figure 3A and 3B, as well as Figure 4A-C, are not cited in the Results text. Please ensure that all panels are explicitly referenced and appropriately discussed.

Reviewer #2: The manuscript presents the computational design and experimental validation of a thermotolerant T7 RNA polymerase variant, named PCD9, utilizing the PROSS web server. To account for the highly dynamic nature of the enzyme during transcription, the authors used four distinct crystal structures representing the promoter, initiation, transition, and elongation complexes as inputs for the PROSS server. Through this multi-state design approach, the authors identified PCD9, a variant that retains full-length transcription activity at 48 °C, a temperature at which the wild-type (wt) enzyme is nearly inactive. Under optimized buffer conditions, PCD9 successfully synthesized kilobase-length transcripts and demonstrated a complete absence of detectable double-stranded RNA (dsRNA) byproducts at 48 °C in a dot blot assay. The study also highlights that PROSS variants derived solely from the elongation complex structure were catalytically inactive, demonstrating the limitations of single-state stability design for flexible enzymes.

However, below are some of the comments/questions I have for the authors to address-

1. The authors did refer to some of the modifications being researched or used in prior studies that talks about how the change in that particular amino acid (or a bunch of amino acids) has changed the functionality of T7 RNA polymerase. I would highly recommend talking about the most important changes in the amino acid configuration of PCD9 and how mechanistically it might have changed the thermostability. I understand that random mutations, sometimes might bring us to the solutions we want, but understanding the core of the solution and linking that to the actual reasons is what matters. For example, some of the mutations resembles with T3 RNA polymerase, another family of viral polymerase. Some of the other ones are in a loop, that might be more tolerable to such mutations (in other words can be redundant).

2. You noted that PCD9 produced visibly weaker dsRNA signals than the wild-type enzyme even at 37 °C, suggesting that the reduction in dsRNA might not rely solely on temperature, but could involve altered reaction kinetics or abortive transcription rates. Can you provide any kinetic data (e.g., Km, Vmax) to support this hypothesis and clarify how the mutations affect baseline activity?

3. The thermal inactivation assay involved pre-incubating the enzymes in a buffer devoid of NTP substrates and DNA template. Because ligand binding often stabilizes proteins, how might the presence of these active transcription components shift the temperature tolerance, and would testing in their presence provide a more accurate reflection of operational stability?

4. When transcribing the kilobase-length template at 48 °C, the TapeStation electropherogram showed a broadened profile with shorter RNA species, which you attribute to potential polymerase pausing and premature termination. Have the authors checked for any sequence specificity of this pause/termination? How significantly does this truncation affect the overall quantitative yield of the full-length therapeutic-grade transcript? Can it be an effect of presence of trace amounts of salts in the purified RNA?

5. No sequence information for the RNA (both short and long) is available. Addgene plasmid number, or sequence information in another format is required. (Ignore it if already submitted in a publicly accessible database that I have missed)

6. It would be interesting to see all the modified protein in a native protein gel electrophoresis and how it compares to wild-type in folding.

**********

6. PLOS authors have the option to publish the peer review history of their article (what does this mean?). If published, this will include your full peer review and any attached files.

If you choose “no”, your identity will remain anonymous but your review may still be made public.

**Do you want your identity to be public for this peer review?**  For information about this choice, including consent withdrawal, please see our Privacy Policy.

Reviewer #1: No

Reviewer #2: No

**********

[NOTE: If reviewer comments were submitted as an attachment file, they will be attached to this email and accessible via the submission site. Please log into your account, locate the manuscript record, and check for the action link "View Attachments". If this link does not appear, there are no attachment files.]

To ensure your figures meet our technical requirements, please review our figure guidelines: https://journals.plos.org/plosone/s/figures

You may also use PLOS’s free figure tool, NAAS, to help you prepare publication quality figures: https://journals.plos.org/plosone/s/figures#loc-tools-for-figure-preparation.

NAAS will assess whether your figures meet our technical requirements by comparing each figure against our figure specifications.

# Author comment: Author response to Decision Letter 1

**DOI:** 10.1371/journal.pone.0353775.r002

25 May 2026

see attachment

Attachment Submitted filename: Response to Reviewers.docx

# Peer review: Decision Letter 1

**DOI:** 10.1371/journal.pone.0353775.r003

**Authors:** Jian Xu

24 Jun 2026

PONE-D-26-10563R1

Engineering of T7 DNA-dependent RNA polymerase with activity at elevated temperature

PLOS One

Dear Dr. Marx,

Thank you for submitting your manuscript to PLOS ONE. After careful consideration, we feel that it has merit but does not fully meet PLOS ONE’s publication criteria as it currently stands. Therefore, we invite you to submit a revised version of the manuscript that addresses the points raised during the review process.

Please submit your revised manuscript by Aug 08 2026 11:59PM. If you will need more time than this to complete your revisions, please reply to this message or contact the journal office at plosone@plos.org. When you're ready to submit your revision, log on to https://www.editorialmanager.com/pone/ and select the 'Submissions Needing Revision' folder to locate your manuscript file.

Please include the following items when submitting your revised manuscript:

- A letter that responds to each point raised by the academic editor and reviewer(s). You should upload this letter as a separate file labeled 'Response to Reviewers'.
- A marked-up copy of your manuscript that highlights changes made to the original version. You should upload this as a separate file labeled 'Revised Manuscript with Track Changes'.
- An unmarked version of your revised paper without tracked changes. You should upload this as a separate file labeled 'Manuscript'.

If you would like to make changes to your financial disclosure, please include your updated statement in your cover letter. Guidelines for resubmitting your figure files are available below the reviewer comments at the end of this letter.

If applicable, we recommend that you deposit your laboratory protocols in protocols.io to enhance the reproducibility of your results. Protocols.io assigns your protocol its own identifier (DOI) so that it can be cited independently in the future. For instructions see: https://journals.plos.org/plosone/s/submission-guidelines#loc-laboratory-protocols. Additionally, PLOS ONE offers an option for publishing peer-reviewed Lab Protocol articles, which describe protocols hosted on protocols.io. Read more information on sharing protocols at https://plos.org/protocols?utm_medium=editorial-email&utm_source=authorletters&utm_campaign=protocols.

As the corresponding author, your ORCID iD is verified in the submission system and will appear in the published article. PLOS supports the use of ORCID, and we encourage all coauthors to register for an ORCID iD and use it as well. Please encourage your coauthors to verify their ORCID iD within the submission system before final acceptance, as unverified ORCID iDs will not appear in the published article. *Only*  the individual author can complete the verification step; PLOS staff *cannot*  verify ORCID iDs on behalf of authors.

We look forward to receiving your revised manuscript.

Kind regards,

Jian Xu, Ph.D.

Academic Editor

PLOS One

Journal Requirements:

If the reviewer comments include a recommendation to cite specific previously published works, please review and evaluate these publications to determine whether they are relevant and should be cited. There is no requirement to cite these works unless the editor has indicated otherwise.

Please review your reference list to ensure that it is complete and correct. If you have cited papers that have been retracted, please include the rationale for doing so in the manuscript text, or remove these references and replace them with relevant current references. Any changes to the reference list should be mentioned in the rebuttal letter that accompanies your revised manuscript. If you need to cite a retracted article, indicate the article’s retracted status in the References list and also include a citation and full reference for the retraction notice.

[Note: HTML markup is below. Please do not edit.]

Reviewers' comments:

Reviewer's Responses to Questions

**Comments to the Author**

1. If the authors have adequately addressed your comments raised in a previous round of review and you feel that this manuscript is now acceptable for publication, you may indicate that here to bypass the “Comments to the Author” section, enter your conflict of interest statement in the “Confidential to Editor” section, and submit your "Accept" recommendation.

Reviewer #1: All comments have been addressed

Reviewer #2: All comments have been addressed

**********

2. Is the manuscript technically sound, and do the data support the conclusions?

The manuscript must describe a technically sound piece of scientific research with data that supports the conclusions. Experiments must have been conducted rigorously, with appropriate controls, replication, and sample sizes. The conclusions must be drawn appropriately based on the data presented.

Reviewer #1: Yes

Reviewer #2: Partly

**********

3. Has the statistical analysis been performed appropriately and rigorously?

Reviewer #1: Yes

Reviewer #2: N/A

**********

4. Have the authors made all data underlying the findings in their manuscript fully available?

The PLOS Data policy requires authors to make all data underlying the findings described in their manuscript fully available without restriction, with rare exception (please refer to the Data Availability Statement in the manuscript PDF file). The data should be provided as part of the manuscript or its supporting information, or deposited to a public repository. For example, in addition to summary statistics, the data points behind means, medians and variance measures should be available. If there are restrictions on publicly sharing data—e.g. participant privacy or use of data from a third party—those must be specified.

Reviewer #1: Yes

Reviewer #2: Yes

**********

5. Is the manuscript presented in an intelligible fashion and written in standard English?

PLOS ONE does not copyedit accepted manuscripts, so the language in submitted articles must be clear, correct, and unambiguous. Any typographical or grammatical errors should be corrected at revision, so please note any specific errors here.

Reviewer #1: Yes

Reviewer #2: Yes

**********

6. Review Comments to the Author

Please use the space provided to explain your answers to the questions above. You may also include additional comments for the author, including concerns about dual publication, research ethics, or publication ethics. (Please upload your review as an attachment if it exceeds 20,000 characters)

Reviewer #1: The revised manuscript is acceptable after a minor correction. Please confirm whether “Figure 1D” on line 190 should be “Figure 1C,” and whether “Figure 1C” on line 205 should be “Figure 1D.” Once this issue is addressed, I recommend acceptance.

Reviewer #2: The authors have provided satisfactory responses to all comments to the best of their ability.

**********

7. PLOS authors have the option to publish the peer review history of their article (what does this mean?). If published, this will include your full peer review and any attached files.

If you choose “no”, your identity will remain anonymous but your review may still be made public.

**Do you want your identity to be public for this peer review?**  For information about this choice, including consent withdrawal, please see our Privacy Policy.

Reviewer #1: No

Reviewer #2: **Yes:** Ruptanu Banerjee

**********

[NOTE: If reviewer comments were submitted as an attachment file, they will be attached to this email and accessible via the submission site. Please log into your account, locate the manuscript record, and check for the action link "View Attachments". If this link does not appear, there are no attachment files.]

To ensure your figures meet our technical requirements, please review our figure guidelines: https://journals.plos.org/plosone/s/figures

You may also use PLOS’s free figure tool, NAAS, to help you prepare publication quality figures: https://journals.plos.org/plosone/s/figures#loc-tools-for-figure-preparation.

NAAS will assess whether your figures meet our technical requirements by comparing each figure against our figure specifications.

# Author comment: Author response to Decision Letter 2

**DOI:** 10.1371/journal.pone.0353775.r004

28 Jun 2026

see file attached

Attachment Submitted filename: Second Response to Reviewers.docx

# Editor report: Decision Letter 2

**DOI:** 10.1371/journal.pone.0353775.r005

**Authors:** Jian Xu

29 Jun 2026

Engineering of T7 DNA-dependent RNA polymerase with activity at elevated temperature

PONE-D-26-10563R2

Dear Dr. Marx,

We’re pleased to inform you that your manuscript has been judged scientifically suitable for publication and will be formally accepted for publication once it meets all outstanding technical requirements.

Within one week, you’ll receive an e-mail detailing the required amendments. When these have been addressed, you’ll receive a formal acceptance letter and your manuscript will be scheduled for publication.

An invoice will be generated when your article is formally accepted. Please note, if your institution has a publishing partnership with PLOS and your article meets the relevant criteria, all or part of your publication costs will be covered. Please make sure your user information is up-to-date by logging into Editorial Manager at Editorial Manager® and clicking the ‘Update My Information' link at the top of the page. For questions related to billing, please contact billing support.

If your institution or institutions have a press office, please notify them about your upcoming paper to help maximize its impact. If they’ll be preparing press materials, please inform our press team as soon as possible -- no later than 48 hours after receiving the formal acceptance. Your manuscript will remain under strict press embargo until 2 pm Eastern Time on the date of publication. For more information, please contact onepress@plos.org.

Kind regards,

Jian Xu, Ph.D.

Academic Editor

PLOS One

Additional Editor Comments (optional):

Reviewers' comments:

# Editor report: Acceptance letter

**DOI:** 10.1371/journal.pone.0353775.r006

**Authors:** Jian Xu

PONE-D-26-10563R2

PLOS One

Dear Dr. Marx,

I'm pleased to inform you that your manuscript has been deemed suitable for publication in PLOS One. Congratulations! Your manuscript is now being handed over to our production team.

At this stage, our production department will prepare your paper for publication. This includes ensuring the following:

* All references, tables, and figures are properly cited

* All relevant supporting information is included in the manuscript submission,

* There are no issues that prevent the paper from being properly typeset

You will receive further instructions from the production team, including instructions on how to review your proof when it is ready. Please keep in mind that we are working through a large volume of accepted articles, so please give us a few days to review your paper and let you know the next and final steps.

Lastly, if your institution or institutions have a press office, please let them know about your upcoming paper now to help maximize its impact. If they'll be preparing press materials, please inform our press team within the next 48 hours. Your manuscript will remain under strict press embargo until 2 pm Eastern Time on the date of publication. For more information, please contact onepress@plos.org.

You will receive an invoice from PLOS for your publication fee after your manuscript has reached the completed accept phase. If you receive an email requesting payment before acceptance or for any other service, this may be a phishing scheme. Learn how to identify phishing emails and protect your accounts at https://explore.plos.org/phishing.

If we can help with anything else, please email us at customercare@plos.org.

Thank you for submitting your work to PLOS ONE and supporting open access.

Kind regards,

PLOS ONE Editorial Office Staff

on behalf of

Dr. Jian Xu

Academic Editor

PLOS One
